# N368-Tau fragments generated by legumain are detected only in trace amount in the insoluble Tau aggregates isolated from AD brain

**DOI:** 10.1186/s40478-019-0831-2

**Published:** 2019-11-13

**Authors:** Kerstin Schlegel, Khader Awwad, Roland G. Heym, David Holzinger, Annika Doell, Stefan Barghorn, Thomas R. Jahn, Corinna Klein, Yulia Mordashova, Michael Schulz, Laura Gasparini

**Affiliations:** 1 Neuroscience Discovery, AbbVie Deutschland GmbH & Co. KG, Knollstrasse, 67061 Ludwigshafen, Germany; 2 Drug Metabolism and Pharmacokinetics, AbbVie Deutschland GmbH & Co. KG, Knollstrasse, 67061 Ludwigshafen, Germany; 3 NBE Analytical R&D, AbbVie Deutschland GmbH & Co. KG, Knollstrasse, 67061 Ludwigshafen, Germany; 4 Discovery and Exploratory Statistics (DIVES), AbbVie Deutschland GmbH & Co. KG, Knollstrasse, 67061 Ludwigshafen, Germany

**Keywords:** Tau protease, Tau truncation, Insoluble Tau, Alzheimer disease

## Abstract

Intraneuronal insoluble inclusions made of Tau protein are neuropathological hallmarks of Alzheimer Disease (AD). Cleavage of Tau by legumain (LGMN) has been proposed to be crucial for aggregation of Tau into fibrils. However, it remains unclear if LGMN-cleaved Tau fragments accumulate in AD Tau inclusions.

Using an in vitro enzymatic assay and non-targeted mass spectrometry, we identified four putative LGMN cleavage sites at Tau residues N167-, N255-, N296- and N368. Cleavage at N368 generates variously sized N368-Tau fragments that are aggregation prone in the Thioflavin T assay in vitro. N368-cleaved Tau is not detected in the brain of *legumain* knockout mice, indicating that LGMN is required for Tau cleavage in the mouse brain in vivo. Using a targeted mass spectrometry method in combination with tissue fractionation and biochemical analysis, we investigated whether N368-cleaved Tau is differentially produced and aggregated in brain of AD patients and control subjects. In brain soluble extracts, despite reduced uncleaved Tau in AD, levels of N368-cleaved Tau are comparable in AD and control hippocampus, suggesting that LGMN-mediated cleavage of Tau is not altered in AD. Consistently, levels of activated, cleaved LGMN are also similar in AD and control brain extracts. To assess the potential accumulation of N368-cleaved Tau in insoluble Tau aggregates, we analyzed sarkosyl-insoluble extracts from AD and control hippocampus. Both N368-cleaved Tau and uncleaved Tau were significantly increased in AD as a consequence of pathological Tau inclusions accumulation. However, the amount of N368-cleaved Tau represented only a very minor component (< 0.1%) of insoluble Tau.

Our data indicate that LGMN physiologically cleaves Tau in the mouse and human brain generating N368-cleaved Tau fragments, which remain largely soluble and are present only in low proportion in Tau insoluble aggregates compared to uncleaved Tau. This suggests that LGMN-cleaved Tau has limited role in the progressive accumulation of Tau inclusions in AD.

## Introduction

Intraneuronal inclusions made of filamentous aggregates of the microtubule-associated protein Tau are hallmarks of Alzheimer disease (AD) and related neurodegenerative diseases termed tauopathies, which include progressive supranuclear palsy, corticobasal degeneration and frontotemporal lobe dementia. In AD, the formation of Tau inclusions progresses with a typical anatomical pattern as demonstrated by the seminal neuropathological work of Braak and Braak [7] that provide the framework for severity staging. In presymptomatic stages of the disease (Stage I and II), Tau inclusions appear in the entorhinal cortex and hippocampus. During stage III and IV, Tau pathology spreads to the occipito-temporal and insular cortices. At Braak stage V-VI, Tau inclusions propagate throughout the entire cortex [7]. Clinically, subtle clinical manifestations or mild cognitive impairment may occur at Braak stage III-IV. AD is clinically symptomatic at Braak stage V-VI.

Tau physiological function is regulated by extensive post-translational modifications (PTMs), including phosphorylation, methylation, nitrosylation and acetylation [25]. In AD brain, aberrant levels and patterns of Tau PTMs are found in insoluble Tau filaments. Among PTMs, Tau truncation by enzymatic cleavage has gained attention during the last decade for its potential pathogenic effects. Tau proteolysis occurs through activity of several proteases, including calpains, cathepsin and caspases, forming variously sized fragments with increased aggregation propensity or neurotoxic properties (see [21] for review). Specifically, the lysosomal protease legumain (LGMN) has been recently reported to cleave Tau and induce the formation of aggregation-prone Tau fragments [29].

LGMN is synthesized as zymogen (pro-LGMN, 56 kDa), which is auto-catalytically processed by sequential removal of C- and N-terminal pro-peptides to generate a 36 kDa active LGMN enzyme at acidic pH [9], displaying cysteine-dependent asparagine endopeptidase (AEP) activity. In AD brain, reports suggest that LGMN translocates from lysosomes to the cytosol and its activation is increased, leading to Tau hyperphosphorylation [5] and truncation by cleavage at asparagine residues 255 and 368 [29]. LGMN-mediated Tau cleavage generates Tau fragments of different size, including N368-cleaved Tau fragments that have increased aggregation propensity in vitro [29]. Notably, the N368 Tau fragment is present in AD brain and colocalizes with Thioflavin S-positive intraneuronal inclusions, suggesting a potential role of N368-cleaved Tau in the pathological aggregation of Tau [29]. However, it remains unclear whether N368-Tau fragments accumulate in cerebral insoluble inclusions of fibrillar Tau during AD pathology development in humans.

Here we report that in vitro, activated LGMN cleaves Tau at four putative sites (N167, N255, N296 and N368), which generate variously sized N368-Tau fragments that are aggregation prone in vitro. N368-cleaved Tau is virtually absent in the brain of *Legumain* (*Lgmn*) knockout mice, indicating that LGMN is required for Tau cleavage in vivo. In human brain samples, levels of N368-cleaved Tau were comparable in the soluble fractions of AD and control hippocampus, while soluble uncleaved Tau was reduced in AD, resulting in increased N368-cleaved Tau/uncleaved Tau ratio. In the sarkosyl-insoluble extracts from AD brain, both uncleaved and N368-cleaved Tau were increased. However, N368-cleaved Tau represented only a very small proportion (< 0.1%) of insoluble uncleaved Tau and the ratio of N368-cleaved Tau over uncleaved Tau was lower in AD than control. Localization and protein levels of activated LGMN were similar in AD and control brain extracts. Overall our data suggest that Tau is physiologically cleaved by LGMN in the mouse and human brain generating N368-cleaved fragments, which remain largely soluble.

## Materials and methods

### Human brain samples

Anonymized brain samples were obtained from The Netherlands Brain Bank (NBB), Netherlands Institute for Neuroscience, Amsterdam (open access: www.brainbank.nl). All Material has been collected from donors for whom a written informed consent for a brain autopsy and the use of the material and clinical information for research purposes had been obtained by the NBB.

Paraffin-embedded temporal cortex, fresh frozen hippocampus and cerebellum from Braak stage I/II control subjects and Braak stage V/VI AD patients were used in the study. Control and AD patients were sex-matched. Postmortem interval (hours ± standard deviation (SD): controls 7.1 ± 1.9; AD 5.4 ± 1.1; *p* < 0.05, two-sample t-test) and age (mean age ± SD: controls 85.7 ± 8.0 years; AD 71.9 ± 8.4 years; *p* < 0.01, two-sample t-test) were higher in controls than AD. Brain pH was similar in the two groups (controls 6.5 ± 0.3; AD 6.4 ± 0.4; *p* = 0.72, two samples t-test). Patient details are shown in Table 1. Only brains from AD patients and control subjects without a clinical history of ischemia and with histopathologically confirmed absence of ischemia, brain infarcts and cerebral amyloid angiopathy, were selected for the study. Sample size was determined based on previous published reports [5, 6, 29].

**Table 1:** Human brain samples: donor information

	Gender	Age [y]	Braak stage	β-amyloid stage	Post-mortem delay [h]	Brain area used
AD	F	79	V	C	4.2	hippocampus
AD	M	77	V	C	5.4	hippocampus
AD	F	74	V	C	6.1	hippocampus
AD	M	61	V	C	4.2	hippocampus
AD	F	70	VI	C	5.2	hippocampus, cerebellum
AD	M	58	VI	C	5.2	hippocampus, cerebellum
AD	M	84	VI	C	8.1	hippocampus, cerebellum
AD	F	72	VI	C	5.6	hippocampus, cerebellum
AD	F	82	VI	C	5.3	hippocampus, cerebellum
AD	M	64	VI	C	4.4	hippocampus, cerebellum
AD	F	70	VI	C	6.1	hippocampus
Control	M	70	I	O	6.2	hippocampus
Control	F	86	I	A	8.1	hippocampus
Control	M	86	I	O	11.5	hippocampus
Control	F	91	I	n.a.	4.5	hippocampus
Control	M	75	I	C	6.2	hippocampus
Control	M	84	I	B	7.2	hippocampus
Control	F	92	I	A	7.5	hippocampus
Control	F	85	II	A	7.5	hippocampus
Control	F	93	II	O	7.4	hippocampus
Control	F	95	II	A	5.2	hippocampus

*n.a.* not available

### Experimental animal models

The following Tau transgenic mice were used in this study: 2.5- and 5.8-month old homozygous Thy1.P301Stau (licensed from the Medical Research Council, UK) [1]; 2- and 5-month old heterozygous rTg4510 mice (licensed from the Mayo Clinic, Jacksonville Florida, USA) [23]. All transgenic mice were bred for Abbvie by Charles River Laboratories (Sulzfeld, Germany). The mice were in a temperature- and humidity-controlled room with a 12:12 h dark/light cycle with ad libitum access to water and food. All animal experiments were performed in full compliance with the Principles of Laboratory Animal Care (NIH publication No. 86–23, revised 1985) in an AAALAC accredited program where veterinary care and oversight was provided to ensure appropriate animal care. All animal studies were approved by the government of Rhineland Platinate (Landesuntersuchungsamt Koblenz) and conducted in accordance with the directive 2010/63/EU of the European Parliament and of the Council on the protection of animals used for scientific purpose, the ordinance on the protection of animals used for experimental or scientific purposes (German implementation of EU directive 2010/63; BGBl. I S. 3125, 3126), the Commission Recommendation 2007/526/EC on guidelines for the accommodation and care of animals used for experimental and other scientific purposes, the German Animal Welfare Act (BGBl. I S. 1206, 1313) amended by Art. 1 G from 17 December 2018 I 2586. rTg4510 mice express 0N4R human P301L mutant hTau under CaMKIIα promoter. rTg4510 mice develop progressive accumulation of sarkosyl-insoluble Tau and AT100 immunoreactive (AT100+) inclusions throughout the forebrain from 4 months of age (SantaCruz et al. 2005). Thy1.P301S Tau mice express 0N4R human P301S mutant Tau under the control of the mouse neuronal Thy.1 promoter. They progressively develop widespread filamentous inclusions made of hyperphosphorylated Tau throughout the cerebral cortex, brain stem, spinal cord and retina, accompanied by neuronal dysfunction, motor deficit and paraparesis [1, 11, 24].

Brain tissue from *Lgmn* knockout (*Lgmn*^−/−^), *Lgmn* heterozygous (*Lgmn*^+/−^) and *Lgmn* wild type (*Lgmn*^+/+^) 2-month old littermates were obtained from Albert-Ludwigs-Universität Freiburg, Germany [18].

### In vitro enzymatic LGMN cleavage assay and non-targeted LC-MS/MS analysis of tau fragments

Recombinant LGMN protein (100 μg/ml; R&D Systems #2199-CY) was activated in 50 mM sodium acetate pH 4.0, 100 mM NaCl for 2 h at 37 °C. Recombinant human 2N4R Tau (3 μM; produced as described in [3]) was reduced with 3 mM DTT by shaking for 15 min at 50 °C. Reduced Tau (2.1 μM) was then incubated with activated LGMN at different molar ratios (LGMN:Tau = 1:5, 1:10, 1:20, 1:100) in PBS pH 6.0, shaking at 850 rpm for 60 min at 37 °C in a total volume of 50 μl. The enzymatic reaction was stopped by addition of 1x Laemmli buffer and heating for 5 min at 95 °C. Protein fragments were separated with Criterion TGX 4–20% SDS-PAGE (Bio-Rad) and stained with Coomassie or transferred to 0.2 μm PVDF membranes (Bio-Rad) for western blot analysis (see below). Anti-N368-cleaved Tau antibody (15,000; Millipore, #ABN1703) was used to identify N368-cleaved Tau fragments.

For non-targeted LC-MS/MS analysis, bands of interest were excised, and in-gel digested with trypsin. In brief, gel bands were reduced with 1.5 mg/mL DTT, alkylated with 10 mg/mL iodacetamide and digested with 6.25 ng/μL trypsin (Promega, #V5111) at 37 °C overnight. Samples were evaporated, reconstituted in 0.1% trifluoroacetic acid, desalted with Zip Tips C18 column (Millipore). After evaporation, samples were resuspended in loading buffer (0.1% formic acid), and injected onto an Acclaim™ PepMap™ nano trap column C18, 2 μm, 0.075 × 20 mm (Thermo Fisher Scientific) with a flow rate of 20 μL/min. After three minutes, the nano trap column was connected with the analytical Acclaim™ PepMap™ column C18, 2 μm, 0.075 × 250 mm (Thermo Fisher Scientific) using a flow rate of 300 nL/min for 90 min at 45 °C. Samples were eluted using the following gradient (A: H2O, 0.1% formic acid; B: acetonitrile, 0.1% formic acid): 0–3 min 2% B, 3–7 min 2–8% B, 7–20 min 8–18% B, 20–35 min 18–25% B, 35–50 min 25–30% B, 50–56 min 30–40% B, 56-60 min 40–90% B, 60–70 min 90% B, 70.0–70.1 min 2% B, 70.1–90 min 2% B. Peptides were ionized with 1.9 kV electrospray ionization voltage by the TriVersa NanoMate® (Advion) and analyzed in data dependent acquisition mode by the LTQ Velos Pro mass spectrometer (Thermo Fisher Scientific). A capillary temperature of 200 °C was applied and acquisition was performed in the mass range of 200–2000 m/z at resolution of 30,000. Precursors were accumulated with an AGC target value of 1E6, a maximum injection time of 500 ms and dynamic exclusion of 30 s in a top-5 method. To identify LGMN cleavage sites on human Tau, raw data were analyzed using the PMI Suite (Protein Metrics).

### Brain tissue homogenization and sarkosyl fractionation

All steps of tissue extraction were carried out on ice or at 4 °C unless stated otherwise. Frozen human or mouse brain tissues were thawed on ice and homogenized in lysate buffer (50 mM Tris, pH 7.4, 150 mM NaCl, 20 mM NaF, 1 mM Na_3_VO_4_, 0.5 mM MgSO_4_, cOmplete protease inhibitor with EDTA (Roche, #11697498001) and PhosSTOP phosphatase inhibitor (Roche, #04906845001) at 5 μl/mg tissue using a cooled bead disruptor (FastPrep-24 5G System, MP Biomedicals). Brain homogenates were frozen in liquid nitrogen and stored at − 80 °C until use.

To separate soluble and insoluble Tau fractions, we used a sarkosyl extraction method to fractionate tau seeds/aggregates of different sizes and diffusibility (Additional file 1: Figure S1; modified from [22]). In brief, 1.2 ml of human brain homogenate was centrifuged at 27,000 x g for 20 min to obtain the supernatant (S1) and the pellet (P1). The P1 pellet was resuspended in 1.2 ml of 10 mM Tris/HCl pH 7.4, 1 mM EGTA, 0.8 M NaCl, 10% sucrose, cOmplete protease inhibitor and PhosSTOP phosphatase inhibitor using an Omni Bead Ruptor (Omni International, Inc) at room temperature and then spun at 27,000 x g for 20 min at 4 °C. The supernatant (S2) was transferred to a new tube. The supernatants S1 and S2 were adjusted to a final concentration of 1% sarkosyl, incubated for 1.5 h at room temperature with 750 rpm shaking and subsequently centrifuged at 150,000 x g for 45 min. The supernatants, SS1 and S3, which contain soluble Tau, were transferred to new tubes, flash frozen in liquid nitrogen and stored at − 80 °C until LC-MS/MS analysis. The corresponding pellets, PS1 and P3, which contain sarkosyl-insoluble Tau, were washed once in 200 μl TBS (25 mM Tris pH 7.4, 3 mM KCl, 140 mM NaCl) and resuspended in 120 μl TBS by vortexing and four sonication pulses (2 s each) with a probe sonicator (Sonoplus, Bandelin) at 35% amplitude. For LC-MS/MS analysis, the extracts were flash frozen and stored at − 80 °C until use. For ELISA, 10 μl of the extracts were mixed with 10 μl Laemmli buffer (Bio-Rad, #161–0737), heated for 5 min at 98 °C, flash frozen and stored at − 80 °C until use.

### Quantification of N368-cleaved and uncleaved tau by targeted LC-MS/MS in mouse and human brain extracts

To quantify N368-cleaved Tau, we developed a targeted LC-MS/MS method, which detects a semi-tryptic peptide spanning from the trypsin cleavage site 354 to the LGMN cleavage site 368. To analyze the respective uncleaved Tau in the same mass spec run, we used a tryptic peptide generated by trypsin cleavage at sites 354 and 369. Casein (0.2% in PBS) was used as surrogate matrix for all dilutions. The sequence of the abovementioned peptides quantified by the targeted mass spectrometry is the same in 3R and 4R tau (by convention the numbering refers to 2N4R Tau). Therefore, the quantification method used for human brain assesses fragments arising from all Tau isoforms. The sequence is also conserved between mouse and human, enabling the determination of endogenous mouse Tau as well. Calibration curves were obtained using the peptides Tau354–369 and Tau354–368 for total brain homogenates or full-length 2N4R Tau and Tau_1–368_ for PS1 and P3 extracts ranging from 5 to 20,000 ng/g. All human Tau peptides used for calibration curves (N369 and N368) were synthesized (GenScript Biotech, Leiden, Netherlands). Quality controls were prepared by spiking 2N4R Tau into pooled samples of rTg4510 PS1 and P3 extracts (Additional file 1: Table S1). Quantification of LGMN-cleaved (Tau354–368) and uncleaved Tau (Tau354–369) was performed in 50 μL of calibration standard, total brain homogenates, SS1, S3, PS1 and P3 Tau.

Calibration standards, mouse brain homogenates and soluble SS1 and S3 extracts were reduced with 12 mM DTT for 30 min, alkylated with 27 mM iodacetamide for 30 min, and digested with 5 ng/mL trypsin/LysC (Promega, #Ax499B) at 60 °C for 1 h. Sarkosyl-insoluble PS1 and P3 extracts were pre-digested with 10 ng/μL trypsin/LysC (Promega, #Ax4999) for 1 h at 60 °C in the presence of 3.2 M Urea supplemented with 0.1% RapiGest (Waters, #18600186), followed by heat denaturation for 20 min at 90 °C before reduction with DTT and alkylation with iodacetamide.

Samples were desalted on a solid phase extraction plate (Waters Oasis HLB 30 μm 10 mg) according to the manufacture’s protocol and analyzed on a Waters TQs mass spectrometer operating in multiple reactions monitoring using the following transitions: Tau 354–369, 527.0 ➔ 401.2, Tau 354–370, 427.0 ➔ 657.4 (monitoring of mis-cleavage) and Tau 354–368, 726.2 ➔ 401.3. Chromatographic separation was achieved on an Acquity HSS T3 C18 column (2.1 × 150 mm, Waters, #186001861) with a flow rate of 250 μL/min and a linear gradient elution starting from 90% water / 10% acetonitrile with 0.1% formic acid to 50% water / 50% acetonitrile with 0.1% formic acid within 10 min.

### In vitro Tau aggregation

Recombinant Tau proteins, including full-length 2N4R Tau, Tau_1–368_ (spanning aa 1–368) and Tau_256–368_ (spanning aa 256–368), were generated by overexpression in E.coli. Purification of all recombinant Tau protein constructs and the in vitro Tau aggregation assay setup have been previously described [3]. In brief, the Tau aggregation assay was performed in 384-well plate format with triplicate samples, incubating Tau protein (40 μM) and Heparin (40 μM; SIGMA, #H3393) at 37 °*C.* Tau aggregation was monitored over time using thioflavin T fluorescence in a Cytation5 plate reader (excitation 440 nm; emission 480 nm).

### Western blot analysis

Total brain homogenates were obtained with 0.5% Triton X-100 (Sigma Aldrich) for 40 min at 6 °C, centrifuged for 15 min at 16.000 x g at 6 °C and the supernatant carefully collected. Samples were mixed with Laemmli sample buffer containing 100 mM DTT, heated for 5 min at 98 °C and 20 μg total proteins per lane were loaded onto 12% Criterion TGX Stain-Free SDS-PAGE gels (Bio-Rad, Cat. No. 567–8045). After the separation, the gel was blotted (TransBlot Turbo Transfer System, Bio-Rad) on a 0.2 μm PVDF membrane (Bio-Rad; Cat.No.170–4157). The membrane was blocked with 5% milk in TBS 0.1% Tween-20 (TBST), incubated with the primary anti-LGMN goat polyclonal antibody (R&D Systems; Cat.No. AF2199) in a 1:200 dilution in 5% milk in TBST followed by HRP-conjugated anti-goat secondary antibody (1:10.000; Jackson, Cat. No. 305–035-045) in 5% milk in TBST. Signals were developed with the Clarity Western ECL Substrate (Bio-Rad, Cat.No. 170–5061) using the ChemiDoc Touch Imaging System (BioRad). To normalize total loaded protein, the blots were stripped using the Restore PLUS Western Blot Stripping Buffer, (Thermo Scientific, Cat. No. 46430) and re-probed using an anti-actin rabbit antibody (1:10.000; Sigma; Cat.No. A5060) in 5% milk in TBST and developed with an HRP-conjugated anti-rabbit IgG antibody (Invitrogen; Cat.No. 32260).

The pro-LGMN and activated-LGMN levels were determined against a standard curve of a pro-LGMN/cleaved pro-LGMN standard-mix which was prepared as follows: recombinant human pro-LGMN (R&D Systems; Cat.No. 2199-CY) was activated at pH 4.0 at 37 °C for 2 h and the activation was stopped by dilution in SDS-PAGE sample buffer with DTT and heating for 5 min at 98 °C. The activation of the pro-LGMN by this method was only partial resulting in a mix of pro-LGMN/activated-LGMN. The in vitro activated-LGMN serves as a surrogate marker for brain activated-LGMN. The recombinant pro-LGMN protein runs at a slightly higher molecular weight than the band detected in human samples; however, this is not critical for the quantification as the relative group comparison was performed on the ratio activated-LGMN/pro-LGMN in AD versus Control brain homogenates. The individual concentrations of pro-LGMN and activated-LGMN were normalized to the individual actin levels to account for potential loading difference of the total protein.

### Immunohistochemistry

Immunohistochemical analysis on Thy1.P301Stau mouse brain was performed on free-floating sections as previously described [4]. Immunohistochemical analysis of rTg4510 mouse brain was performed using paraffin-embedded sections. In brief, animals were anesthetized with Ketamin/Xylazin and trans-cardially perfused with PBS. Hemibrains were immersion-fixed for 24 h at RT, and transferred to 70% EtOH. Tissue was trimmed and samples dehydrated using standard EtOH series followed by Xylene and paraffin embedding (ASP300, Leica). 4 μm Paraffin sections were prepared and processed on a Leica BOND Rx automated stainer using citrate-buffer based heat-induced antigen retrieval solution (Epitope Retrieval Solution 1 Bond, Leica, Germany) and DAB-based detection (Leica, Germany). Tau pathology was detected using AT100 anti-pS212/pT214 Tau conformational antibody (MN1060; Thermo Scientific) [2].

For analysis of human brain paraffin-embedded samples, 4 μm sections were processed on a Leica Bond RX automated stainer using citrate buffer-based heat-induced antigen retrieval for both DAB-based and fluorescence-based detection. The following antibodies were used: AT100 antibody for Tau pathology; anti-beta amyloid 6E10 antibody (Biolegend); anti-LAMP2 (Invitrogen); anti-legumain antibody clone 11B7 (kindly provided by Dr. C. Watts, University of Dundee, UK) [16]. Images have been collected using the a 3DHistec P1000 scanner for bright field images or a 3DHistec Midi scanner for fluorescence.

### Statistical methods

Experimental data were analyzed using two-sample t-test, paired t-test, Welch’s test, Wilcoxon test, one-way analysis of variances (ANOVA) followed by post-hoc tests presuming normal or lognormal underlying distribution or further transformed data (see Additional file 1: Table S2 for details). To explore the underlying distribution of the data, the Shapiro-Wilk test was performed to assess normality and, if needed, data were transformed into logarithm or other transformation. After transformation, the homogeneity of variances was assessed using Levene’s test. One-way ANOVA analyses were followed by Dunnett’s post-hoc test, test contrast or Tukey’s HSD for honest significant difference. Non-parametric tests were used when the assumption of normality was violated. A summary of all statistical assumptions and tests is presented in the Additional file 1: Table S2. Statistical analyses were carried out with Graph Pad Prism (Version 7.00), JMP 13.1.0 (SAS institute) Software**.** Exact *p* values are reported in figure legends. In the figure plots, for simplicity, statistical significance is indicated by * and ** stars for *p* < 0.05 and *p* < 0.01, respectively.

## Results

### LGMN cleaved Tau at sites N167, N255, N296 and N368 in vitro

**Fig. 1:**
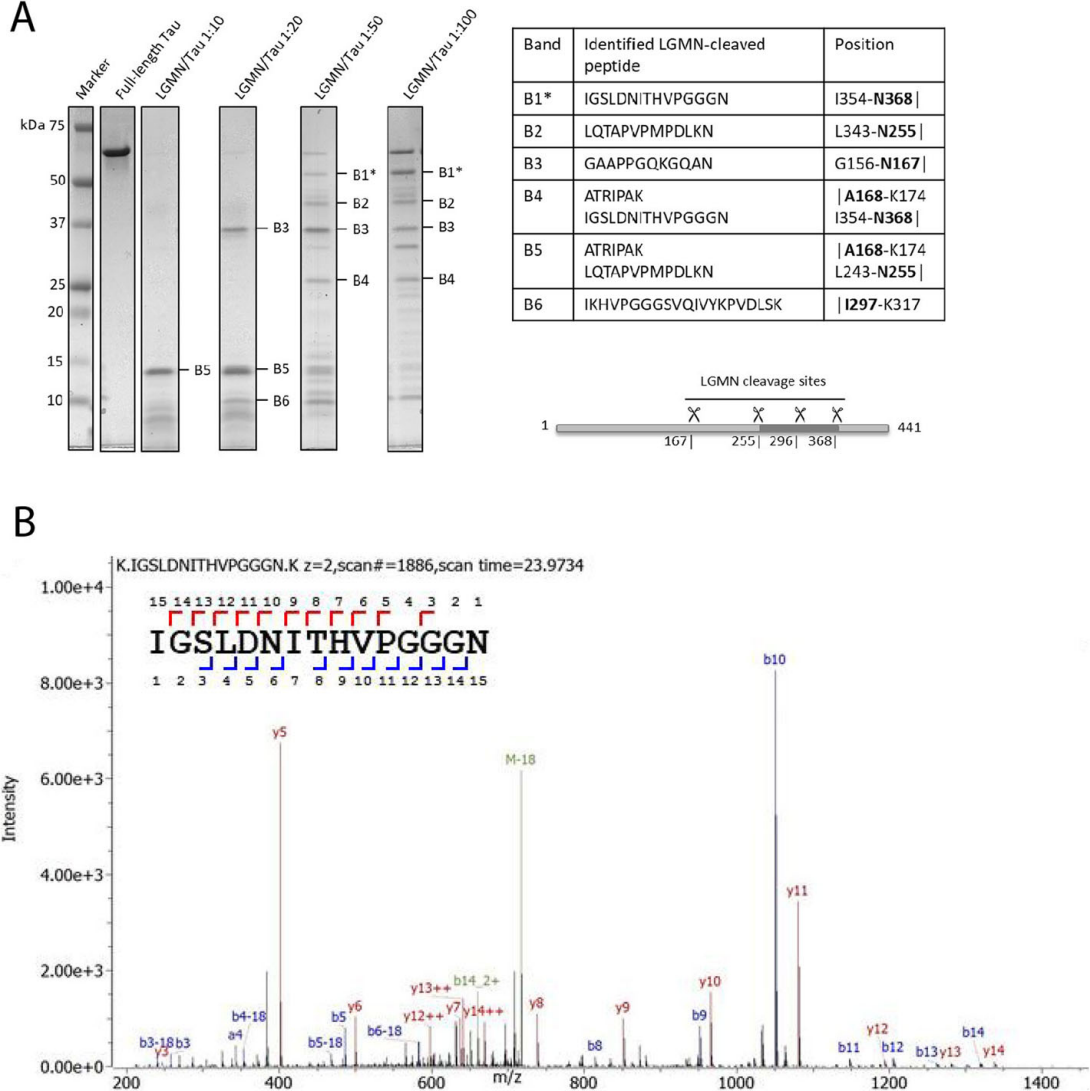
LGMN cleaves human Tau in vitro. **a**. Coomassie stained gels of Tau fragments after enzymatic digestion with LGMN in vitro. Tau and LGMN were incubated in vitro at different molar ratios and the cleaved fragments were resolved by SDS-PAGE. Bands B1-B6 were analyzed by LC-MS/MS after in-gel tryptic digestion. Identified LGMN-cleaved peptides are indicated in the table. Four LGMN cleavage sites were identified at residues N167, N255, N297 and N368 as indicated in the Tau scheme. Tau amino acid numbering relates to the 2N4R Tau isoform. In the table, the vertical line (│) indicates LGMN cleavage site. (*) LC-MS/MS for band B1 was performed in a separate experiment. **b**. Fragment spectrum of the peptide IGSLDNITHVPGGGN (amino acids 354–368). Numbers in the peptide sequence refer to the b-ions (top) and y-ions (bottom), respectively

**Fig. 2:**
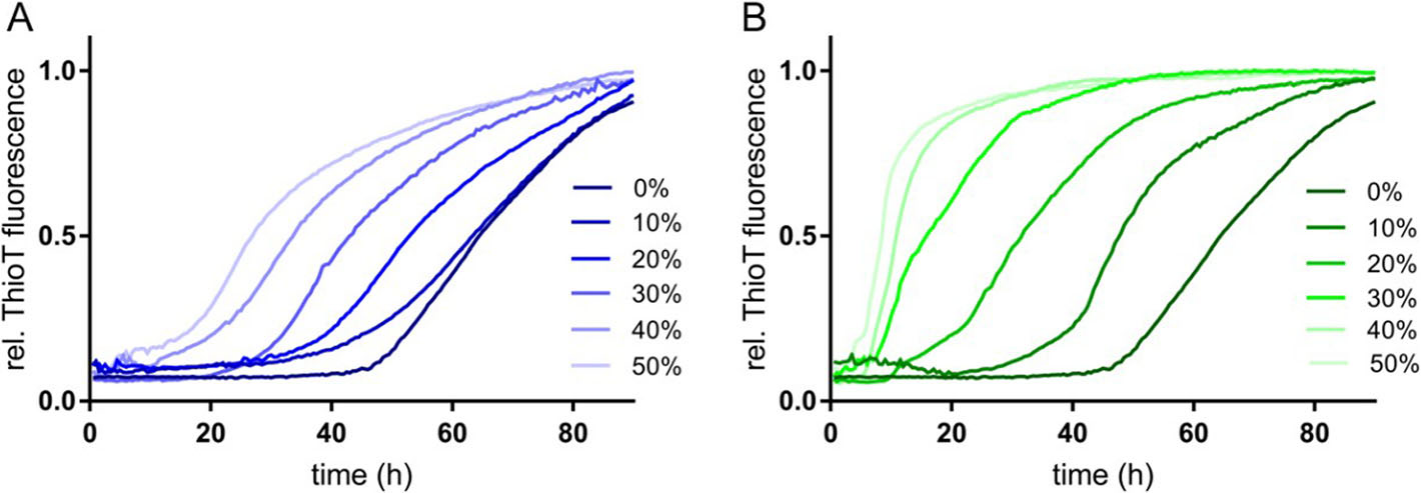
N368-cleaved Tau fragments accelerate the aggregation of full-length Tau in vitro. A-B. Thioflavin S aggregation assays of mixtures between recombinant 2N4R wild-type full-length Tau and recombinant Tau_1–368_(**a**) or Tau_256–368_(**b**) fragments. The amount of Tau fragments in the reaction is indicated as molar fraction percentage respect to full-length Tau (mean, *n* = 3)

To investigate LGMN-mediated Tau cleavage, we incubated recombinant 2N4R human Tau with recombinant LGMN at different molar ratios in vitro. The resulting Tau fragments were initially analyzed by SDS-PAGE and Coomassie gel stain. Full-length Tau ran at an apparent molecular weight of 60 kDa. Upon incubation with LGMN, at increasing substrate concentration, up to 6 major bands (bands B1-B6) were detected (Fig. 1a). Using in-gel digestion with trypsin followed by non-targeted LC-MS/MS analysis of Tau bands, we identified five semi-tryptic peptides that could be assigned to LGMN cleavage after four asparagine residues: N167 in the mid-domain of Tau, N255 in the R1 repeat, N296 in the R2 repeat, and N368 at the end of the R4 repeat (Fig. 1a). Western blot analysis using an antibody that preferentially recognizes N368-cleaved Tau further confirmed the presence of N368-cleaved Tau fragments running at the same size as bands B1, B4 and in bands of lower molecular weight (Additional file 1: Figure S2). Cleavage of Tau at N368 is of interest as it generates Tau fragments that have been previously shown to be aggregation-prone in vitro [29]. Indeed, in the thioflavin T aggregation assay, two possible recombinant N368-Tau fragments, the longest Tau_1–368_ and the shortest Tau_256–368_, accelerated the aggregation of full-length 2N4R Tau when incubated at different molar ratios (Fig. 2). Considering the potential role in Tau aggregation, in all subsequent analyses, we therefore focused on quantification of N368-cleaved Tau.

These results indicate that LGMN cleaves Tau in vitro at four distinct sites forming differently sized Tau fragments, including aggregation-prone N368-Tau fragments.

### LGMN cleaved endogenous mouse Tau in vivo

**Fig. 3:**
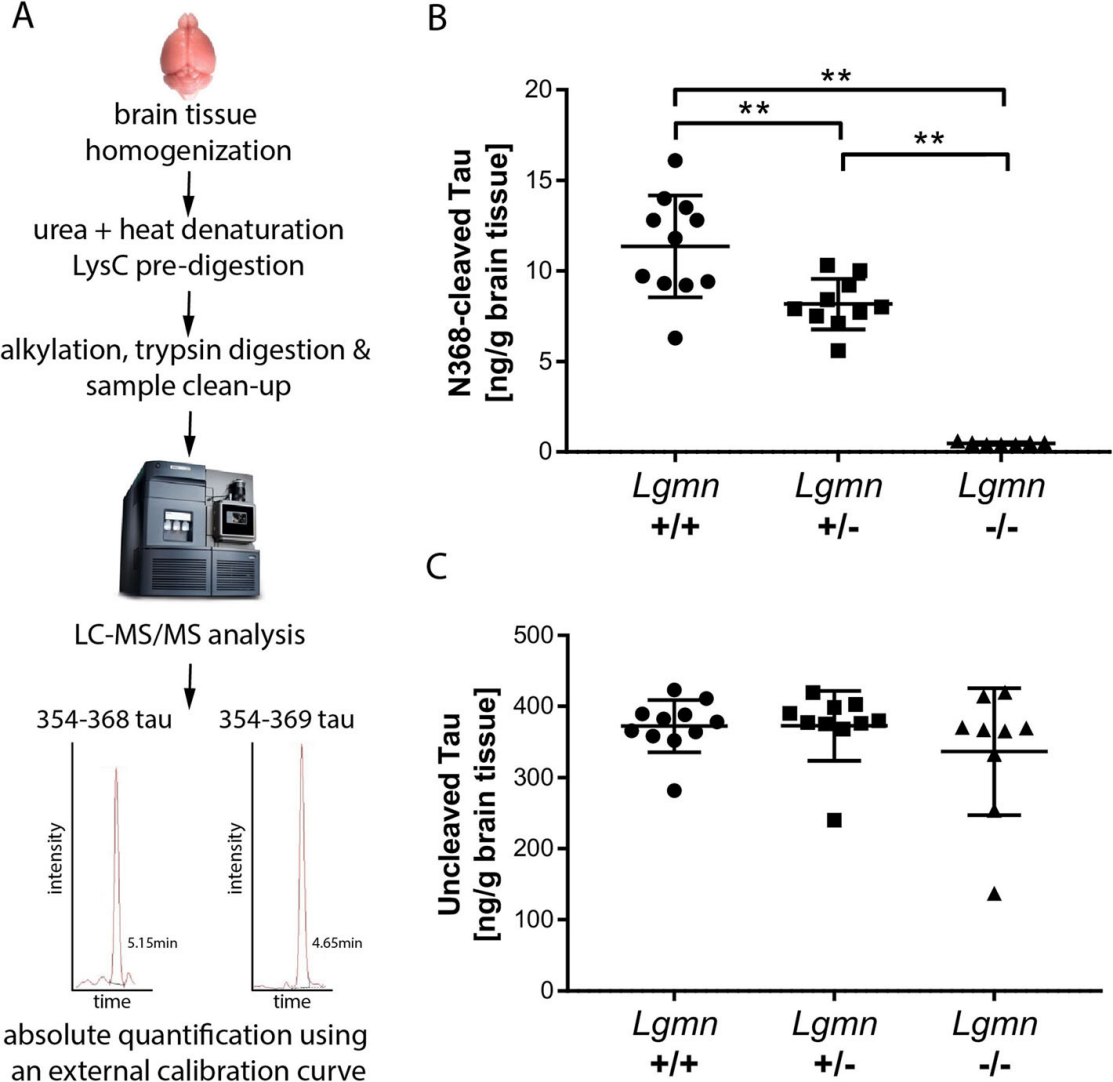
N368-cleaved Tau is not detected in *Lgmn* knockout mouse brain total homogenate. **a**. Scheme of the workflow for the quantification of Tau from mouse total brain extracts using LC-MS/MS. **b-c**. Mass spectrometry quantification of 354–368 (**b**) and 354–369 (**c**) Tau peptides as a mesure of N369-cleaved and uncleaved Tau, respectively, in forebrain tissue from *Lgmn*^−/−^ (*n* = 9), *Lgmn* ^+/−^ (*n* = 10) and *Lgmn*^+/+^ (*n* = 11) littermates at 2 months of age. **b**. Levels of N368-cleaved Tau are significantly lower in *Lgmn* ^+/−^ compared to *Lgmn*^+/+^ mice (*p* = 0.0070) and are below LLOQ in *Lgmn*^−/−^ (*p* < 0.0001 vs *Lgmn*^+/−^ and *Lgmn*^+/+^). **c**. Levels of uncleaved Tau were similar across genotypes (*p* = 0.153 *Lgmn*^+/−^ vs *Lgmn*^−/−^; *p* = 0.494 *Lgmn*^+/+^ vs *Lgmn*^−/−^; *p* = 0.597 *Lgmn*^+/−^ vs *Lgmn*^+/+^). In all graphs, data points, mean and standard deviation are shown. **p < 0.01

To investigate whether LGMN cleaves Tau in vivo, we analyzed N368-cleaved Tau in the forebrain of *Lgmn*^−/−^ mice compared to heterozygous (*Lgmn*^+/−^) and wild-type (*Lgmn*^+/+^) mice by targeted LC-MS/MS (Fig. 3a). We quantified N368-cleaved Tau by measuring the semi-tryptic 354–368 Tau peptide (354 and 368 are trypsin and LGMN cleavage sites, respectively). Uncleaved Tau was quantified by measuring the tryptic peptide 354–369. N368-cleaved Tau was detected in decreasing amount in *Lgmn*^+/+^, *Lgmn*^+/−^ and *Lgmn*^−/−^ mice, displaying significant gene-dose dependent reduction of N368-cleaved Tau (Fig. 3b). In *Lgmn*^+/−^ brain, N368-cleaved Tau was 28% lower than in *Lgmn*^+/+^. In *Lgmn*^−/−^, N368-cleaved tau levels were below the lower limit of quantification. Levels of uncleaved Tau were similar across all genotypes (Fig. 3c). These data indicate that LGMN cleaves Tau in the mouse brain in vivo and suggest that LGMN is the main protease cleaving Tau at N368.

### N368 Tau did not change in total homogenate of tau transgenic mouse brain at different pathology stages mice

**Fig. 4:**
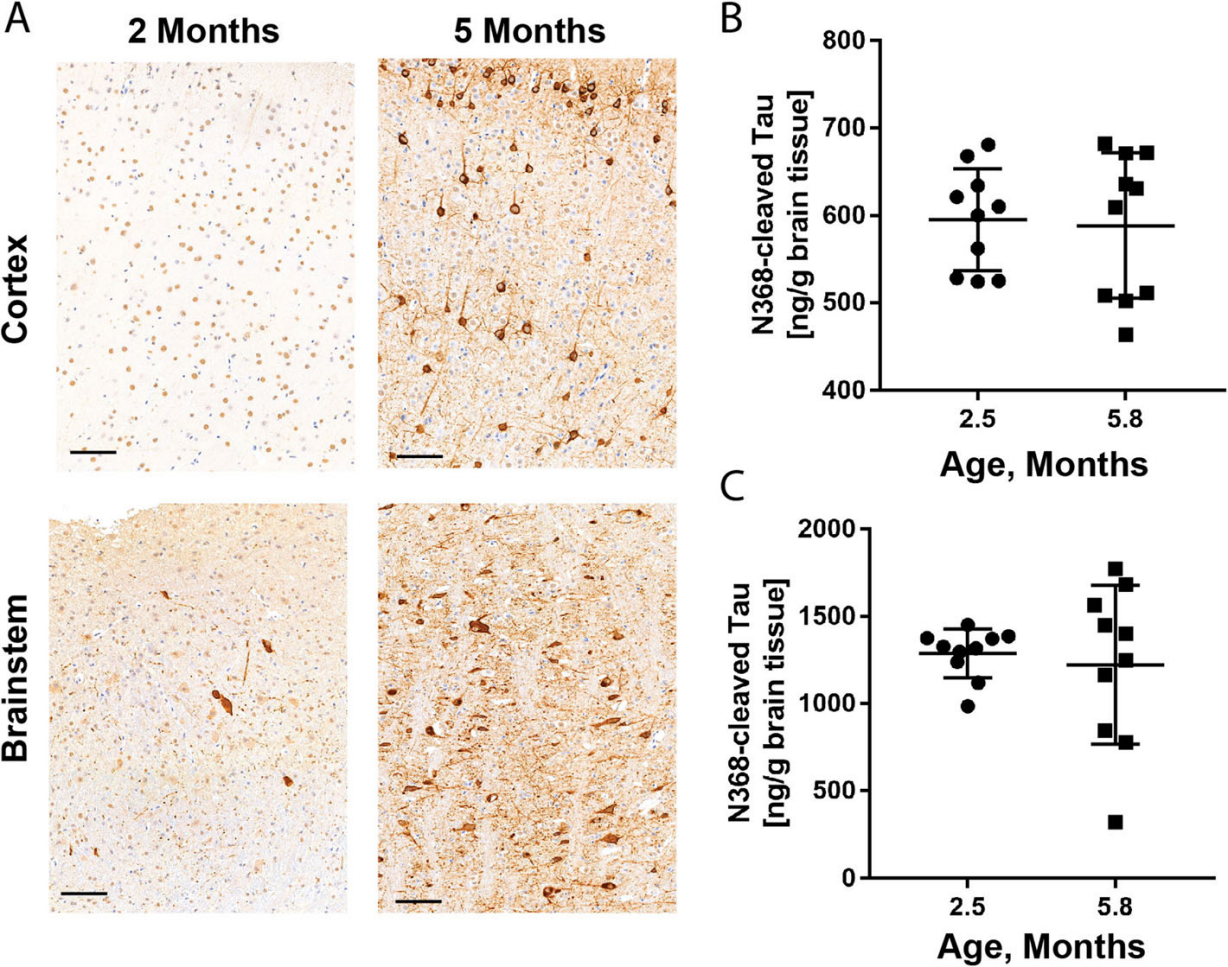
Total levels of N368-cleaved Tau do not change in the total homogenate brain of Tau transgenic mice. **a**. Insoluble Tau inclusions in the brainstem and cortex of Thy1.P301STau transgenic mice at 2 and 5 months of age. Tau inclusions are immunostained using the phosphorylation- and conformation-dependent AT100 antibody. Scale bars: 100 μm. **b, c**. In total homogenate of Thy1.P301STau transgenic cortex (**b**) and brainstem (**c**), N368-cleaved Tau levels are similar at 2.5 and 5.8 months of age (cortex, *p* = 0.675; brainstem, *p* = 0.850; *n* = 10/group). The quantification of Tau in the total mouse brain homogenate was performed by LC-MS/MS as depicted in Fig. 3a. In all graphs, data points, mean and standard deviation are shown

We next sought to investigate whether total levels of N368-cleaved Tau increase during the development of Tau pathology in two transgenic mouse models of tauopathy, the Thy1.P301Stau [1] and the rTg4510 [23] mice. In Thy1.P301Stau mice, levels of N368-cleaved Tau were higher in the homogenate from brainstem than cortex (Fig. 4). In both brain areas, N368-cleaved Tau levels were similar at 2.5 months of age, when AT100 immunoreactive (AT100^+^) Tau inclusions are sparse, and 5.8 months of age, when AT100^+^ Tau inclusions are abundant [1]. Similar results were obtained in total homogenate of rTg4510 mice. The ratio of N368-cleaved Tau over uncleaved Tau was reduced in the cortex of rTg4510 mice at 5 months of age, when AT100^+^ tangle-like inclusions are abundant, compared to 2 month old mice, which have only limited AT100^+^ Tau inclusions (Additional file 1: Figure S3) [23].

These data indicate that N368-cleaved Tau is produced at similar levels at different stages of development of Tau inclusions in the mouse brain and is independent of the pathological aggregation of Tau.

### N368 Tau levels were comparable in the soluble extracts of AD and control brain

**Fig. 5:**
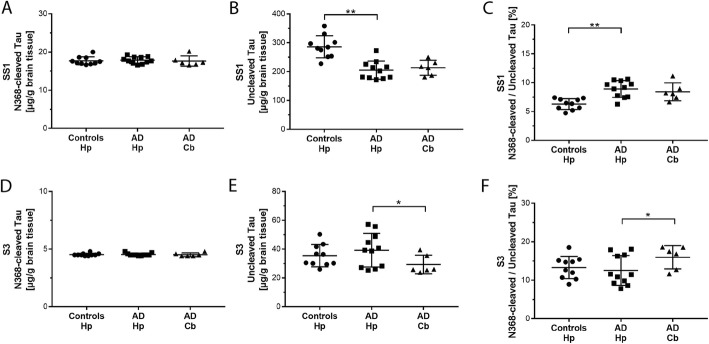
Soluble levels of N368-cleaved Tau are similar in AD and control hippocampus. Soluble Tau extracts SS1 and S3 from human hippocampus and cerebellum were prepared as described in the Methods. Levels of N368-cleaved ad uncleaved Tau were measured by LC-MS/MS. **a.** In the sarkosyl-soluble SS1 extracts, levels of N368-cleaved-Tau are comparable in AD and control hippocampus (*p* = 0.654) and AD hippocampus vs cerebellum (*p* = 0.310). **b.** Levels of uncleaved-Tau are significantly lower in the sarkosyl-soluble SS1 extracts of human AD than control hippocampus (*p* < 0.0001). Uncleaved Tau is similar in AD hippocampus and cerebellum (*p* = 0.380). **c.** In the sarkosyl-soluble SS1 extracts, the percentage N368-cleaved Tau over uncleaved Tau is significantly higher in AD than control hippocampus (*p* = 0.0001) and is similar in AD hippocampus and cerebellum (*p* = 0.304). **d.** In the sarkosyl-soluble S3 extracts, levels of N368-cleaved Tau are comparable in AD hippocampus vs. control hippocampus (*p* = 0.970) or AD hippocampus vs. cerebellum (*p* = 0.296). **e.** In the sarkosyl-soluble S3 extracts, levels of uncleaved Tau are comparable in AD vs. control hippocampus (*p* = 0.454). In AD, uncleaved Tau is significantly lower in the cerebellum than hippocampus (*p* = 0.030). **f.** In the sarkosyl-soluble S3 extracts, the percentage N368-cleaved Tau over uncleaved Tau is comparable in AD vs control hippocampus (*p* = 0.505). In AD, N368-cleaved Tau percentage is significantly higher in cerebellum vs hippocampus (*p* = 0.033). AD hippocampus, *n* = 11; AD cerebellum, *n* = 6; Control hippocampus, *n* = 10. Hp, hippocampus; Cb, cerebellum. In all graphs, data points, mean and standard deviation are shown. *p < 0.05; **p < 0.01

We next investigated whether N368-cleaved Tau is differentially produced in AD and control brains. Using targeted LC-MS/MS, we quantified the N368-cleaved Tau in the sarkosyl-soluble SS1 and S3 extracts (Additional file 1: Figure S1 and Methods) of hippocampus from Braak stage I/II control subjects and Braak stage V/VI AD patients (Fig. 5). For AD cases, we also analyzed levels of N368-cleaved Tau in the cerebellum, which is typically unaffected by AD pathology. The majority of uncleaved Tau and N368-cleaved Tau was detected in the SS1 soluble fraction, where the abundance of uncleaved Tau (Fig. 5b, e) and N368-cleaved Tau (Fig. 5a, d) was about 7-fold and 4-fold higher than in S3 soluble extracts, respectively. In all extracts, levels of uncleaved Tau measured by LC-MS/MS positively correlated to levels of total Tau quantified using a Tau12/HT7 ELISA (Additional file 1: Figure S4; Additional file 1: Figure S5) validating the robustness of our targeted LC-MS/MS method. In both SS1 and S3 soluble extracts, the amounts of N368-cleaved Tau were comparable in control and AD hippocampus and AD cerebellum (Fig. 5a, d). In AD hippocampus, uncleaved Tau was reduced compared to controls (Fig. 5b; Suppl. Fig. 4), leading to a significant increased percentage of cleaved Tau over uncleaved in AD (8.9% in AD vs 6.3% in controls; Fig. 5c). Levels of uncleaved Tau were similar in AD hippocampus and cerebellum, resulting in a similar ratio of N368-cleaved over uncleaved Tau (8.4% in the cerebellum; Fig. 5b-c). In the S3 soluble extracts, levels of N368-cleaved and uncleaved Tau were similar in control and AD hippocampus (Fig. 5d-e) and the percentage of N368-cleaved Tau over uncleaved was 12.6 and 13.3%, respectively (Fig. 5f). In AD cerebellum, cleaved Tau represented 16.0% due to lower levels of uncleaved Tau in this pathology-free brain area compared to hippocampus (Fig. 5f).

Overall, these data indicate that *i)* N368-cleaved Tau is rather abundant in the soluble extracts of human hippocampus and cerebellum; *ii)* its levels are comparable in the hippocampus of AD and controls; iii) in AD, N368-cleaved Tau levels do not differ in brain areas bearing Tau pathology, such as hippocampus, compared to cerebellum, which is typically devoid of AD pathology.

### N368 Tau was present at trace amounts in the sarkosyl-insoluble extracts of AD brain

**Fig. 6:**
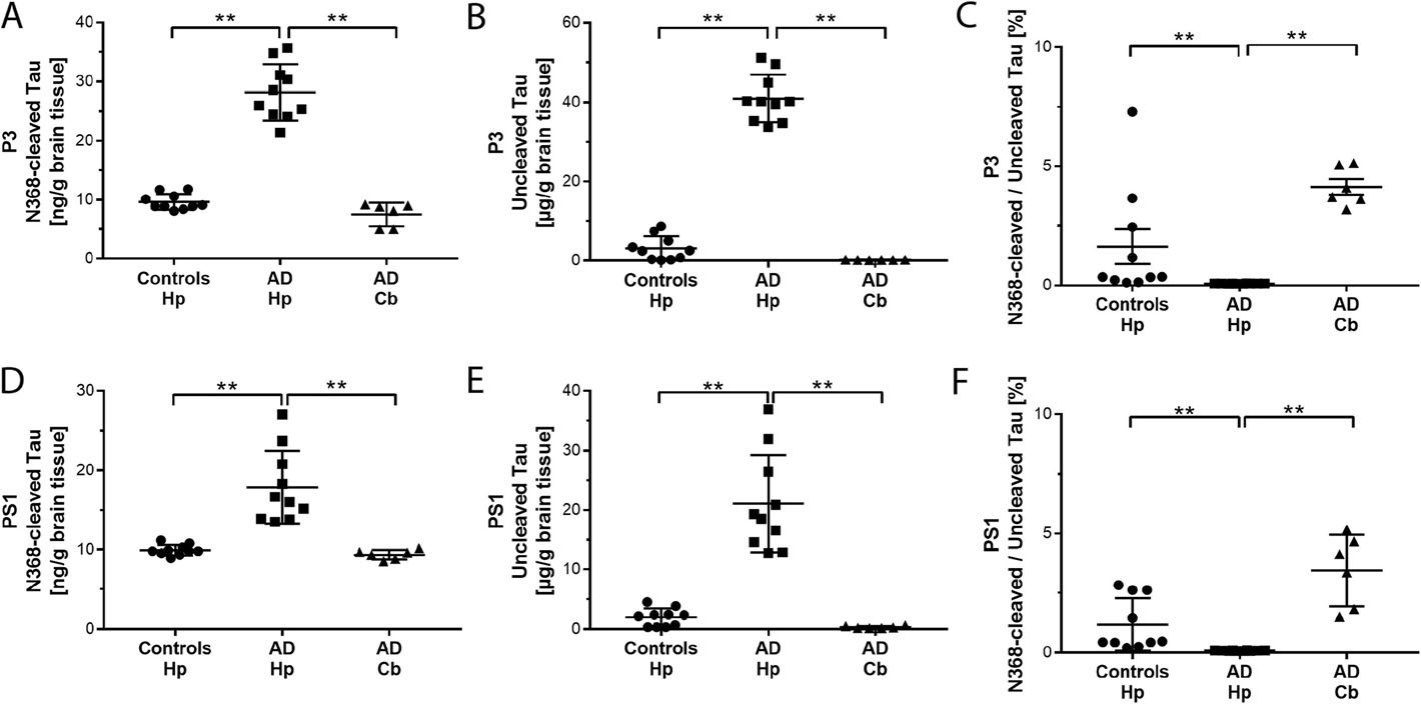
Insoluble levels of N368-cleaved and uncleaved Tau are higher in AD than control hippocampus. Insoluble Tau extracts P3 and PS1 from human hippocampus and cerebellum were prepared as described in the Methods. Levels of N368-cleaved ad uncleaved Tau were measured by LC-MS/MS. **a-b**. In the sarkosyl-insoluble P3 extracts, levels of both N368-cleaved (**a**) and uncleaved Tau (**b**) are significantly higher in AD hippocampus compared to control hippocampus (*p* < 0.0001) and AD cerebellum (**a**, *p* < 0.0001; **b**, *p* = 0.0003). **c**. In the sarkosyl-insoluble P3 extracts, the percentage N368-cleaved Tau over uncleaved Tau is significantly lower in AD hippocampus than control hippocampus (*p* = 0.001) and AD cerebellum (*p* = 0.001). **d-e**. In the sarkosyl-insoluble PS1 extracts, levels of both N368-cleaved (**d**) and uncleaved Tau (**e**) are significantly higher in AD hippocampus compared to control hippocampus (*p* < 0.0001) and AD cerebellum (**d**, *p* = 0.002; **e**, *p* = 0.001). **f**. In the sarkosyl-insoluble PS1 extracts, the percentage N368-cleaved Tau over uncleaved Tau is significantly lower in AD than control hippocampus (*p* < 0.0001) and AD cerebellum (*p* = 0.009). AD hippocampus, *n* = 10; AD cerebellum, *n* = 6; Control hippocampus, *n* = 10. Hp, hippocampus; Cb, cerebellum. In all graphs, data points, mean and standard deviation are shown. **p < 0.01

Considering the pro-aggregation properties of N368-Tau fragments in vitro reported by others [29] and confirmed by our work (Fig. 2), we hypothesized that such fragments may drive the aggregation of Tau in AD and preferentially accumulate into insoluble Tau inclusions. To test this hypothesis, we quantified both the N368-cleaved and uncleaved Tau in the sarkosyl-insoluble extracts, P3 and PS1, of AD and control brains (Fig. 6).

N368-cleaved Tau was present in both the sarkosyl-insoluble P3 and PS1 extracts of AD and control hippocampus and AD cerebellum. In AD hippocampus, N368-cleaved Tau was significantly increased by 2.4- and 1.7-fold in P3 and PS1 extracts, respectively, as compared to control hippocampus (Fig. 6a, d). In AD cerebellum, levels of N368-cleaved Tau were comparable to control hippocampus. In both P3 and PS1-extracts, uncleaved Tau was increased by about 8-fold in AD hippocampus compared to controls and was very low in AD cerebellum (Fig. 6b, e). Despite the apparent substantial increase, in AD hippocampus, N368-cleaved Tau represented only 0.07 and 0.09% of uncleaved Tau in P3 and PS1 sarkosyl-insoluble extracts, respectively. These values are significantly lower than the percentages found in the P3 and PS1 extracts from control hippocampus (1.6% in P3 and 1.3% in PS1) or AD cerebellum (4.1 and 3.5% for P3 and PS1, respectively) (Fig. 6c, f).

Overall, these data do not support our initial hypothesis and indicate that despite being present in the sarkosyl-insoluble extracts of human brain, the N368-cleaved Tau fragments is not the main component of AD insoluble Tau inclusions.

### Protein localization and levels of activated LGMN were comparable in AD and control brains

**Fig. 7:**
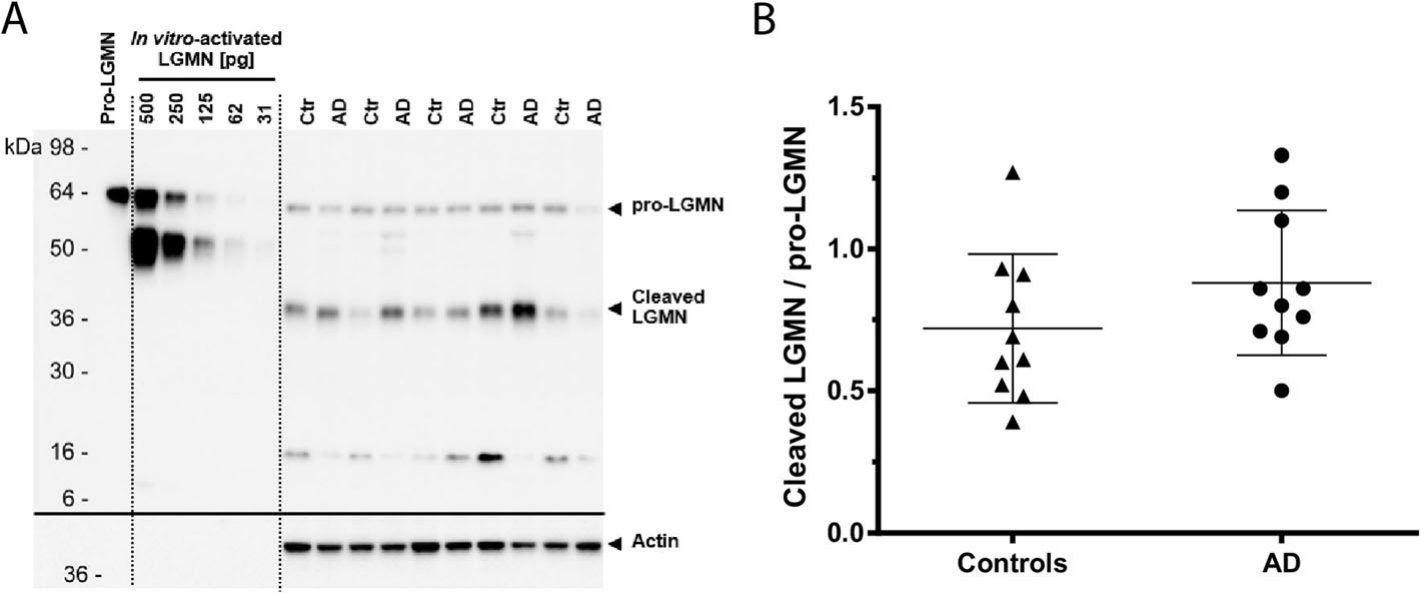
Ratios of cleaved, activated LGMN over pro-LGMN are similar in total homogenate of AD and control hippocampus. Pro-LGMN and cleaved, activated LGMN were analyzed in total homogenate by western blot. **a.** Western blot of LGMN protein expression in AD and control (Ctr) hippocampus. Recombinant human pro-LGMN (316 pg) and in vitro-activated LGMN are loaded as reference. Equal proteins of Triton-X brain homogenate were loaded for each sample. Actin was used as loading control. **b.** Ratio of cleaved LGMN/pro-LGMN. AD hippocampus, *n* = 10; Control hippocampus, *n* = 10. In all graphs, data points, mean and standard deviation are shown. *p* = 0.182

Previous findings showed that LGMN activation is increased in AD brain, likely due to mislocalization [5, 29]. However, our data indicate that in both AD and controls, levels of N368-cleaved Tau are similar in the soluble fractions, which contain the vast majority of Tau (Additional file 1: Figure S4; Figure S5B, E; Figure S6B, E), and are increased only in the insoluble fractions, where uncleaved Tau levels are also elevated (Fig. 6a, d). We therefore investigated whether LGMN was differentially activated in AD and control brains. LGMN is synthesized as zymogen (pro-LGMN, 56 kDa) and is auto-catalytically processed by sequential removal of N- and C-terminal pro-peptides to generate a 36 kDa active LGMN enzyme [8]. Using quantitative western blot of pro-LGMN and activated LGMN, we found that in total homogenate, the ratios of activated-LGMN over pro-LGMN were similar in AD and control hippocampus (Fig. 7), indicating that LGMN activity is not altered in AD brain.

**Fig. 8:**
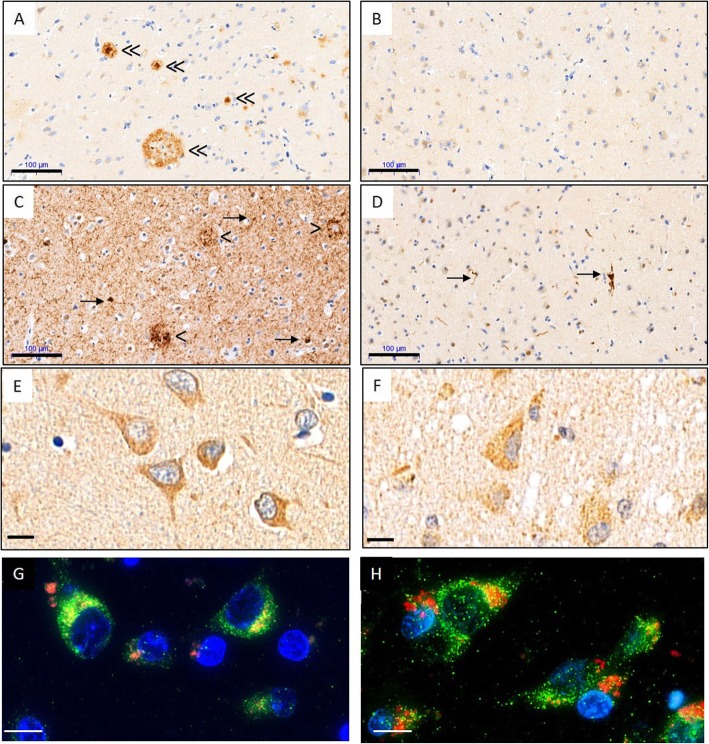
Localization of LGMN is similar in AD and control temporal cortex. Immunohistochemistry analysis of middle temporal gyrus sections from Alzheimer disease (**a**,**c**,**e**,**g**) and non-demented control (**b**, **d**, **f**, **h**) subjects. Adjacent sections were used, and images represent matched fields of view. β-amyloid plaques (double arrowhead) were detected in AD (**a**), but not control (**b**). Abundant AT100 immunoreactive neuritic plaques (arrow head), neurofibrillary tangles (arrows) and neuropil threads (open arrow) were detected in AD (**c**). Sparse neurofibrillary tangles and neuropil threads were also detected in controls (**d**). LGMN immunoreactivity and subcellular distribution was comparable in AD (**e**, **g**) and control samples (**f**, **h**). LGMN (red) and colocalizes with LAMP2 (green) in both AD and controls (**g**, **h**). Scale bars: **a-d**, 100 μm; **e-f**, 20 μm; **g-h**, 10 μm

We finally investigated LGMN localization in the temporal cortex of patients with AD and age-matched control subjects. As expected, abundant β-amyloid plaques, Tau inclusions, Tau immunoreactive neuritic plaques and neuropil threads were detected in AD but not control brains (Fig. 8). In both control and AD cortex, LGMN immunoreactivity was detected in neurons and diffusely throughout the brain parenchyma. In neuronal soma, the immunoreactivity appears punctate, consistent with a vesicular localization of the enzyme. In immunofluorescence analysis, LGMN immunoreactivity partially colocalized with LAMP2 immunoreactive puncta. However, the pattern of LGMN immunoreactivity was comparable in AD and control brains, indicating an overall preservation of the subcellular localization of LGMN (Fig. 8).

## Discussion

Emerging evidence indicates that enzymatic cleavage of Tau plays a key role in the pathogenesis of tauopathy due to the formation of aggregation-prone and/or neurotoxic fragments. Recently, LGMN has been studied due to its ability to affect Tau by a dual mechanism: i) cleaving Tau and generating fragments that are aggregation-prone in vitro ([29] and Fig. 2); ii) inhibiting protein phosphatase 2A (PP2A) through cleavage of its inhibitor 2 (I_2_^PP2A^), leading to Tau hyperphosphorylation [5]. Our findings now indicate that LGMN cleaves Tau in vitro and in the mouse brain in vivo. LGMN cleavage generates variously sized fragments, including N368-Tau fragments that are aggregation-prone in vitro. N368-cleaved Tau is detected in human brain. In soluble extracts of both AD and control hippocampus, N368-cleaved Tau is present at about 6–9% of the level of full-length, uncleaved Tau. However, in the sarkosyl-insoluble tau aggregates from AD brain, N368-cleaved Tau represents only a very minor proportion (< 0.1% of uncleaved Tau). We also find that levels of both pro-LGMN and activated, cleaved LGMN are similar in AD and control brain extracts.

Tau fragmentation may play a key role in the development of Tau pathology and an increasing number of Tau fragments of unknown provenance have been observed in biochemical and proteomic studies [21]. LGMN cleaves Tau at multiple asparagine residues. We find that LGMN cleaves Tau at N255, N368, N167 and N296 in vitro. Both N255- and N368-cleavage have been previously reported in the mouse brain [29]. However, only N167- and N368-cleaved Tau have been detected in human brain [6, 29]. The N255- and N296-Tau fragments have not been detected in human brain samples [6], suggesting that only sites N167 and N368 are accessible to LGMN activity in the human brain.

LGMN has been implicated in the brain physiological and pathological cleavage of Tau. Indeed, consistent with previous findings [29], our data in *Lgmn*^*−/−*^ mouse brain show that LGMN is required for the cerebral production of N368-Tau in vivo. In AD, LGMN-mediated Tau cleavage have been proposed as a key driver of Tau pathology development due to the high aggregation propensity of N368-cleaved tau fragments. Our findings do not support this pathological hypothesis and indicate that N368-cleaved tau fragments, despite having high aggregation propensity in vitro in presence of heparin, remain largely soluble and do not accumulate in insoluble Tau aggregates in AD brain. We think the discrepancy between in vitro and in vivo findings arises from biological, rather than methodological factors.

Indeed, to validate our method, we compared the levels of uncleaved Tau measured by our LC-MS method with quantification of Total Tau by ELISA. The correlation between the two types of measurements was high in all the fractions (Additional file 1: Figure S5), including the sarkosyl-insoluble fractions which are particularly challenging due to potentially inefficient digestion of the aggregated species. The precision of the LC-MS methods in the sarkosyl-insoluble extracts was > 80% (Additional file 1: Table S1). Importantly, we quantify both peptides (N368 and N369) in the same run on the same extracts. Thus, any methodological limitations, e.g. inefficient digestion or extraction, would affect the measurement of both uncleaved and cleaved Tau.

The discrepancy between in vitro and in vivo aggregation behavior of N368-cleaved Tau can be explained by various biological factors. First, the thioflavin T aggregation assay used to study Tau aggregation in vitro is a non-physiological system using recombinant wild-type or mutant human Tau protein and polyanions. Indeed, recombinant Tau protein incubated alone in vitro has very little intrinsic propensity to aggregate [19, 20], possibly due to the lack of post-translational modifications that are present in vivo [10]: Addition of polyanionic cofactors, such as heparin, RNA, fatty acids or fatty acid-like molecules (e.g., arachidonic acid and alkyl sulfonate detergents) is required to trigger its assembly into filaments in vitro [12, 14, 15, 27]. It is unlikely that these crude in vitro aggregation paradigms faithfully predict the aggregation behavior of Tau or its fragments in the brain in vivo, cautioning on the risk of overinterpreting data from such simplified systems to generate pathological hypothesis.

Second, in living neurons, subcellular localization and compartmentalization of active LGMN and LGMN-cleaved products may limit the impact of N368-cleaved Tau fragments on Tau intraneuonal assembly. In previous studies, LGMN translocation from lysosome to cytosol, increased levels of activated LGMN and increased activity of the enzyme have been reported in the brain of AD patients [5, 6, 29]. Unlike previous work, our findings indicate that localization and protein levels of both pro- and activated-LGMN are comparable in the brain of AD and control subjects. Consistently, we also find that the levels of N368-cleaved Tau detected in the soluble extracts, which contain the majority of Tau, are similar in AD and control. This indicates that overall LGMN activity and compartmentalization are unaltered in AD brain, likely preventing the accumulation of N368-Tau in cytoplasmic neuronal inclusions.

There is consensus in the field that a small amount of pathological Tau aggregates may act as ‘seeds’ to initiate, in a prion-like manner, the massive aggregation of monomeric Tau. In principle, the trace amount of N368-cleaved present in the sarkosyl-insoluble Tau extracts could be consistent with a role as seeds initiating the aggregation of Tau. However, the presence at such low amounts can also be a consequence of co-aggregation with uncleaved tau which seems the main component of aggregates. Indeed, uncleaved Tau is increased about 10-fold in AD brain compared to control, while the cleaved form is increased only by 1.5-2x in aggregates from AD brain. This suggests that after the initial seeding event, uncleaved Tau might drive the increasing accumulation of Tau inclusions during Tau pathology progression. In a pilot experiment in an in vivo seeding model in rTg4510 mice, we found that when injected in the CA1 hippocampus of 2-month old rTg4510, both recombinant pre-formed fibrils made of full-length Tau and AD brain extract induced robust AT100-positive Tau pathology in the ipsilateral CA1, while Tau_256–368_ aggregates did not (data not shown). These findings do not support the hypothesis that Tau256–368 aggregates may acts as seeds in vivo. However, more extensive investigation is required to clarify the role of various N368-cleaved Tau fragments as potential aggregation initiators.

In our study, despite a trend to an increased ratio of cleaved LGMN over the pro-enzyme, the levels of cleaved, activated LGMN in AD hippocampus were similar to those of controls. This differs from previous studies from other groups [5, 6, 29], which reported either increased levels of cleaved, activated LGMN [5, 6] or enhanced LGMN activity in AD [29]. This discrepancy may arise from several factors, including specific experimental conditions, type of samples and cohort features.

Total tissue homogenate was obtained by different extraction methods (e.g. presence and concentration of detergent). Levels of cleaved, activated LGMN was evaluated by western blot in our work and previous studies [5, 6], while Zhang and colleagues [29] report results from a LGMN activity assay. In this and previous reports, group sizes are comparable. However, the variability of the measure apparently differs across studies, likely due to type of brain areas considered or cohort inclusion criteria.

The type of brain tissue samples differs among studies. AD and control brains [29] or frontal cortex from neuropathologically confirmed AD and control subjects [5] were used previously. In such studies, no information on Braak and amyloid staging is provided. Recently, Behrendt and colleagues [6] reported LGMN activation in the enthorinal cortex from early AD patients (Braak III-IV) versus controls (Braak 0-I). In our study we analyzed LGMN activation in the hippocampus of late AD (Braak V-VI) versus controls subjects (Braak I-II). Neurodegenerative changes associated with late AD may have hampered our ability to detect significant differences between groups.

The specific features of our cohort may also account for the discrepancy with previous findings. One feature of our study is that we adopted rigorous inclusion criteria that led to an imbalance in subject age: control subjects are significantly older than controls. Considering that ischemic conditions and brain infarcts may trigger LGMN activation [17], only brains from AD patients and control subjects with no clinical history of stroke, hypoxia or ischemia and absence of ischemic lesions, brain infarcts and cerebral amyloid angiopathy at neuropathology were selected for our study. As other studies do not specify the inclusion criteria for selected patient and control brain samples, we cannot exclude that previously reported differences among AD and controls may arise from the confounding presence of ischemic lesions.

## Conclusions

Previous reports have implicated LGMN as the key protease involved in the pathogenesis of AD, frontotemporal dementia and Parkinson disease due to its ability to cleave proteins accumulating in pathological inclusions typical of these diseases, such as Tau, α-synuclein and TDP-43 [13, 26, 28, 29] among many other substrates [8]. In AD, LGMN overactivation and LGMN-mediated Tau cleavage have been proposed as key drivers of Tau pathology development due to high aggregation propensity of LGMN-generated N368-cleaved tau fragments. Overall, our findings do not support this hypothesis and indicate that LGMN physiologically cleaves Tau in the mouse and human brain generating N368-Tau fragments, which remain largely soluble and do not preferentially accumulate in insoluble Tau aggregates. This and the suggested role of LGMN in microglia-mediated degradation of Tau [6] argue against the potential use of LGMN inhibitors as modifying therapy for Tau pathological aggregation.

## Supplementary information

**Additional file 1.** Supplemental Methods, **Table** **S1**-**S2** and **Figure** **S1**-**S6**.

## Data Availability

The datasets supporting the conclusions of this article are included within the article and its supplemental material.
