# Supplementary material for: N368-Tau fragments generated by legumain are detected only in trace amount in the insoluble Tau aggregates isolated from AD brain
Source: Acta Neuropathol Commun. 2019 Nov 13;7:177. doi: 10.1186/s40478-019-0831-2 (PMC6854719; doi:10.1186/s40478-019-0831-2)
Supplement: Supplementary file 1 — Additional file 1. Supplemental Methods, Table S1-S2 and Figure S1-S6. [file 40478_2019_831_MOESM1_ESM.pdf]

N368-Tau fragments generated by legumain asparagine endopeptidase are detected only in trace amount in the insoluble Tau aggregates isolated from AD brain.

Kerstin Schlegel[1]*, Khader Awwad[2]*, Roland Heym[1]*, David Holzinger[1], Annika Doell[3], Stefan Barghorn[1], Thomas Jahn[1], Corinna Klein[1], Yulia Mordashova[4], Michael Schulz[2] and Laura Gasparini[1]

[1]AbbVie Deutschland GmbH & Co. KG, Neuroscience Discovery, [2]AbbVie Deutschland GmbH & Co. KG, Drug Metabolism and Pharmacokinetics; [3]AbbVie Deutschland GmbH & Co. KG, NBE Analytical, [4]AbbVie Deutschland GmbH & Co. KG, Discovery and Exploratory Statistics (DIVES), Knollstrasse, 67061 Ludwigshafen, Germany.

*Equal contribution

Supplemental Material

Supplemental Method

Table S1

Table S2

Figure S1

Figure S2

Figure S3

Figure S4

Figure S5

Figure S6

Supplemental Method

Tau ELISA in sarkosyl extracts of human brain tissue

Tau in human brain sarkosyl extracts was quantified with an ELISA using Tau-12 (BioLegend, # 806502) as capture antibody and Biotin-HT7 (Thermo Scientific # MN1000B) as detection antibody. 2N/4R Tau (Sigma-Aldrich, #T0576) was used as calibrator. For plate coating with the capture antibody, 100µl/well of 2 µg/ml Tau-12 in PBS with 20% Glycerol was adsorbed to Maxisorp NUNC-Immuno Plates (Thermo Scientific, #442404) overnight at 4°C. All following steps were performed at room temperature. The wells were blocked with 2% BSA in PBST and 20% Glycerol for 2h. Calibrators (15.6-1000 pg/ml) and samples were diluted in PBST with 0.1% BSA and applied in duplicates. Plates were incubated for 2 h. After rinsing in PBST, 0.2 µg/ml Biotin-HT7 was added and incubated for 1h. 0.05 µg/ml Streptavidin Poly-HRP (Thermo Scientific, # 21140) in 0.1% BSA in PBST was subsequently added for 1 h, followed by 100 µl/well of TMB substrate (KEM-EN-TEC Diagnostics, #4800A) for 10 min. The reaction was stopped by addition of 100 µl 0.18 M sulphuric acid and the absorbance was measured at 450 nm in a Ledetect 96 plate reader (Anthos Mikrosysteme GmbH).

Table S1 – Spike-in validation experiment for the quantification of Tau 354-369 in the PS1 and P3 sarkosyl-insoluble brain fractions.

Fraction	Spike Amount (ng/mL)	Mean (ng/mL)	Standard deviation (ng/mL)	Coefficient of Variation (%)	Precision (%)
PS1	0	0	9	7	
PS1	245	245*	30	12	87
PS1	1000	830*	47	6	87
PS1	7500	6441*	205	3	83
P3	0	0	349	2	
P3	245	218*	19	9	86
P3	1000	899*	48	5	92
P3	7500	5964*	86	1	79

Indicated levels of recombinant human Tau (2N4R) were spiked to pools of PS1 or P3 fractions from Tg4510 brains and analyzed by mass spectrometry as described in the Material and Method section. *Baseline corrected levels, Tau354-369 level in PS1 Tau levels were 135 ng/mL and in P3 17413 ng/mL of Tg4510 mice.

Table S2 – Statistical appendix

Figure[§]	*Normality*	*Transformation*	*Homogeinity of variances*	*Stats method used*
3B	*Yes (after transformation) Shapiro-Wilk p=0.4441, p=0.6431, p=0.0835*	*Log-transformation*	*Yes (Levene's p=0.1686)*	*One-way ANOVA followed by Tukey's HSD test*
3C	*No (non-param test used)*	*No*	*Yes (Levene's p=0.1041)*	*Wilcoxon each pair test*
4A	*Yes (after transformation)*	*Box-Cox transformation*	*No (Levene's p=0.0090)*	*Welch's test*
4B	*No (non-param test used)*	*No*	*No (Levene's p=0.0144)*	*Kruskall-Walis test*
5A	*Yes (after transformation) Shapiro-Wilk's p=0.1798; p=0.7779*	*Log transformation*	*Yes (Levene's p=0.4218)*	*Two-samples t-test*
	Paired differences (after transformation) are normally distributed Shapiro-Wilk's p=0.1222	*Power transformation*		*Paired t-test*
5B	*Yes, Shapiro-Wilk's p=0.1238, p=0.2455*	*No*	*Yes (Levene's p=0.1067)*	*Two-samples t-test*
	Paired differences are normally distributed, Shapiro-Wilk's p=0.7974	*No*		*Paired t-test*
5C	*Yes (after transformation), Shapiro-Wilk's p=0.2800, p=0.6940*	*Log transformation*	*No (Levene's p<0.0001)*	*Welch's test (unequal variances)*
	Paired differences(after transformation) are normally distributed, Shapiro-Wilk's p=0.9132	*Log transformation*		*Paired t-test*
5D	*Yes (after transformation), Shapiro-Wilk's p=0.0.7612, p=0.2735*	*Log transformation*	*No (Levene's p=0.0026)*	*Welch's test (unequal variances)*
	Paired differences (after transformation) are normally distributed, Shapiro-Wilk's p=0.6136	*Log transformation*		*Paired t-test*
5E	*Yes (after transformation), Shapiro Wilk's test p=0.1076, p=0.3488*	*Power transformation*	*Yes (Levene's p=0.3336)*	*Two-samples t-test*
	Paired differences are normally distributed, Shapiro-Wilk's p=0.7245	*No*		*Paired t-test*

5F	*Yes (after transformation), Shapiro-Wilk's p=0.0545, p=0.5219*	*Log transformation*	*No (Levene's p<0.0001)*	*Welch's test (unequal variances)*
	Paired differences are normally distributed, Shapiro-Wilk's p=0.4243	*No*		*Paired t-test*
6A	*Yes (after transformation) Shapiro-Wilk's p=0.0851; p=0.6540*	*Log transformation*	*Yes (Levene's p=0.8302)*	*Two-samples t-test*
	Paired differences (after transformation) are normally distributed, Shapiro-Wilk's p=0.2981	*Log transformation*		*Paired t-test*
6B	*Yes, Shapiro-Wilk's p=0.9187, p=0.1407*	*No*	*Yes (Levene's p=0.7206)*	*Two-samples t-test*
	Paired differences are normally distributed, Shapiro-Wilk's p=0.8878	*Log transformation*		*Paired t-test*
6C	*Yes (after transformation), Shapiro-Wilk's p=0.3692, p=0.2113*	*Log transformation*	*Yes (Levene's p=0.6883)*	*Two-samples t-test*
	Paired differences(after transformation) are normally distributed, Shapiro-Wilk's p=0.3847	*No*		*Paired t-test*
6D	*No, Shapiro-Wilk's p=0.0325, p=0.0191*	*No*	*Yes (Levene's p=0.8307)*	*Wilcoxon's test (non-param)*
	Paired differences are normally distributed, Shapiro-Wilk's p=0.8201	*No*		*Paired t-test*
6E	*Yes (after transformation), Shapiro Wilk's test p=0.4601, p=0.2485*	*Power transformation*	*Yes (Levene's p=0.2081)*	*Two-samples t-test*
	Paired differences (after transformation) are normally distributed, Shapiro-Wilk's p=0.2668	*Log transformation*		*Paired t-test*
6F	*Yes (after transformation), Shapiro-Wilk's p=0.7944, p=0.1820*	*Log transformation*	*Yes(Levene's p=0.1871)*	*Two-samples t-test*
	Paired differences (after transformation) are normally distributed, Shapiro-Wilk's p=0.4876	*Log transformation*		*Paired t-test*
7B	*Yes, Shapiro Wilk's p= 0.5263, p=0.5855*	*No*	*Yes (Levene's p=0.8979)*	*Two-samples t-test*
Suppl.3 A	*Yes*	*No*	*Yes (Levene's p=0.1683)*	*Two sample t-test*
Suppl. 4A	*Yes (Shapiro-Wilk's test p=0.1455, p=0.5526, p=0.7471)*	*No*	*Yes (Levene's p=0.9693)*	*One-way ANOVA followed by Dunnett's test*

Suppl. 4B	*Yes (Shapiro-Wilk's test p=0.2851, p=0.2938, p=0.9376)*	*Log transformation*	*Yes (Levene's p=0.0849)*	*One-way ANOVA followed by Dunnett's test*
Suppl. 4C	*Yes (Shapiro-Wilk's test p=0.3647, p=0.8023, p=0.7516)*	*Power transformation*	*Yes (Levene's p=0.1095)*	*One-way ANOVA followed by Dunnett's test*
Suppl. 4D	*No (non-param test used)*	*No*	*No*	*Steel test*

§All graphs are data *scatterplot with mean ±SD*

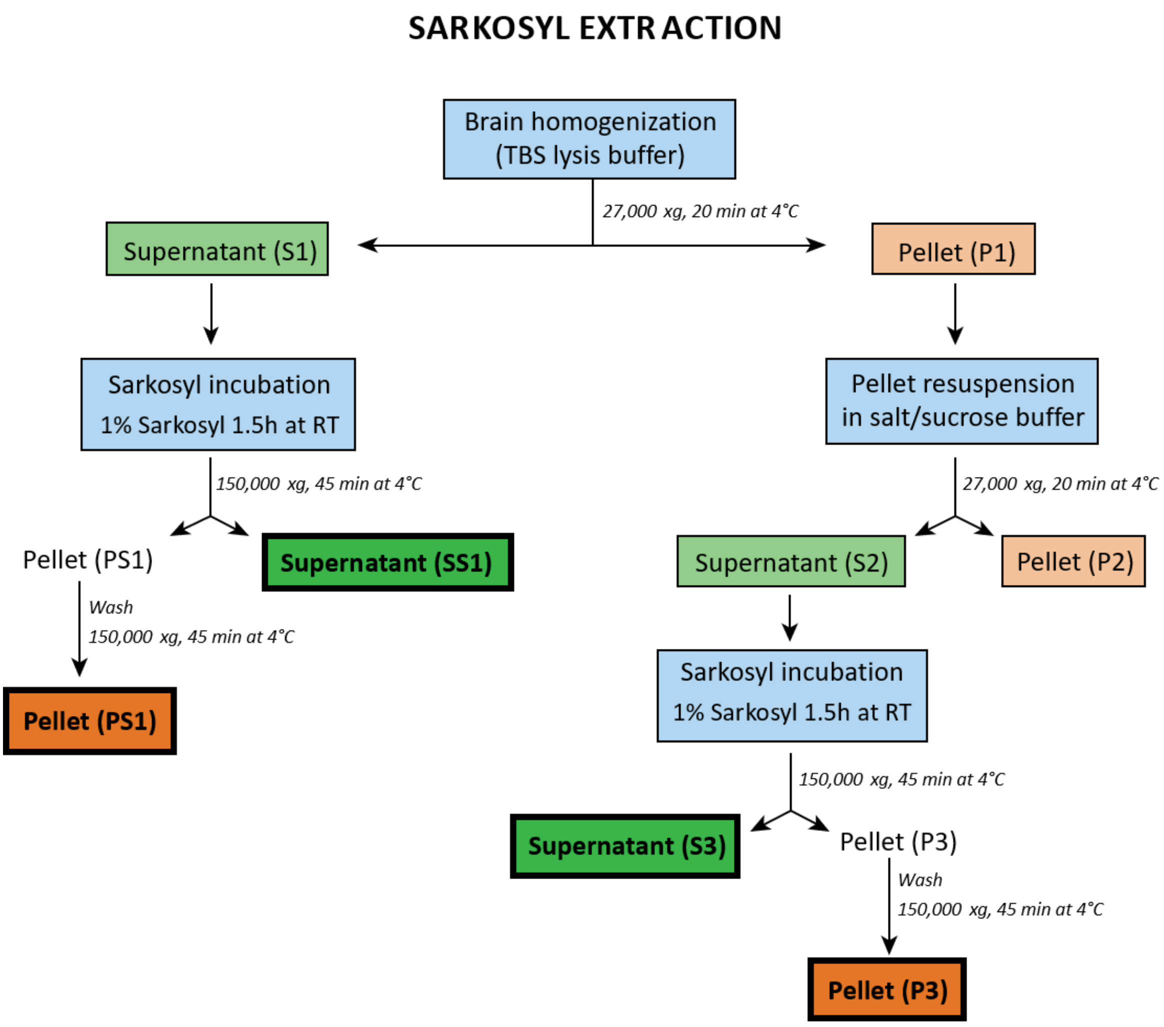

Figure S1 – Scheme of the sarkosyl extraction method.

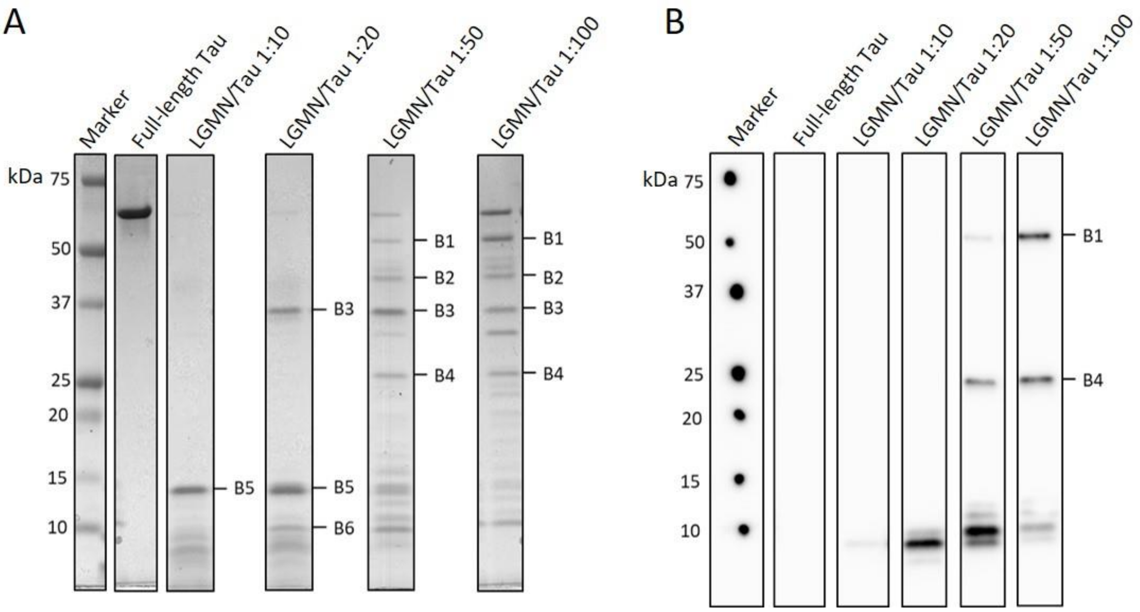

Figure S2 – A. Coomassie stained gels of Tau fragments after enzymatic digestion with LGMN in vitro. Tau and LGMN were incubated in vitro at different molar ratios and the cleaved fragments were resolved by SDS-PAGE (the same gel is shown in Figure 1A). Bands B1 and B4 were confirmed by LC-MS/MS to contain Tau fragments containing amino acids x-N368 (Figure 1A). **B**. Western blot of cleaved Tau as detected by the anti N368-cleaved Tau antibody (Millipore, ABN1703). B1 and B4 bands are recognized by the N368-cleaved Tau antibody.

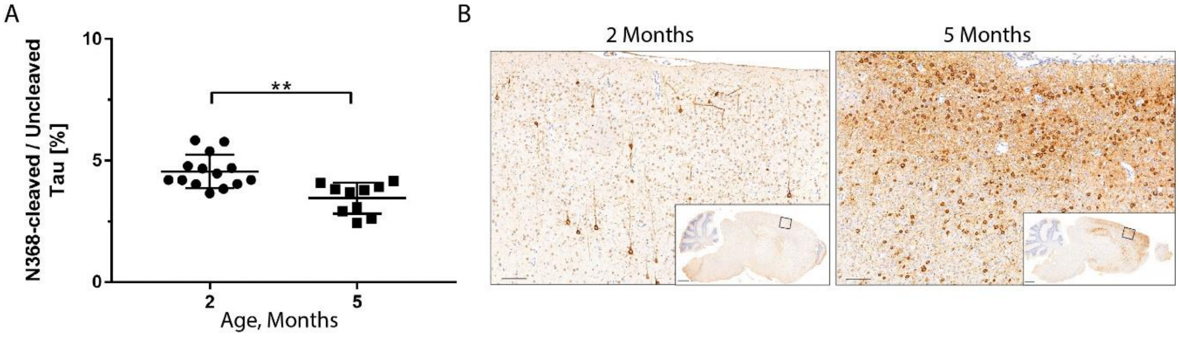

Figure S3 – The abundance of N368-cleaved Tau is reduced in total homogenate of cortex of 5 month-old rTg4510 mice. The quantification of Tau in the total mouse brain homogenate was performed by LC-MS/MS as depicted in Figure 3A. **A.** Percentage of cleaved-Tau over uncleaved Tau in total homogenate of rTg4510 cortex at 2 and 5 months of age (p=0.0007) as determined by targeted LC-MS/MS. In the graph, data points, mean and standard deviation are shown. **p<0.01. **B.** Insoluble Tau inclusions in the cortex of rTg4510 transgenic mice at 2 and 5 months of age. Tau inclusions are immunostained using AT100 antibody. Images in insets represent respective whole brain sagittal section and squared areas represent regions shown in the main panel at higher magnification. Scale bars: 100 μm in main panels; 1 mm in insets.

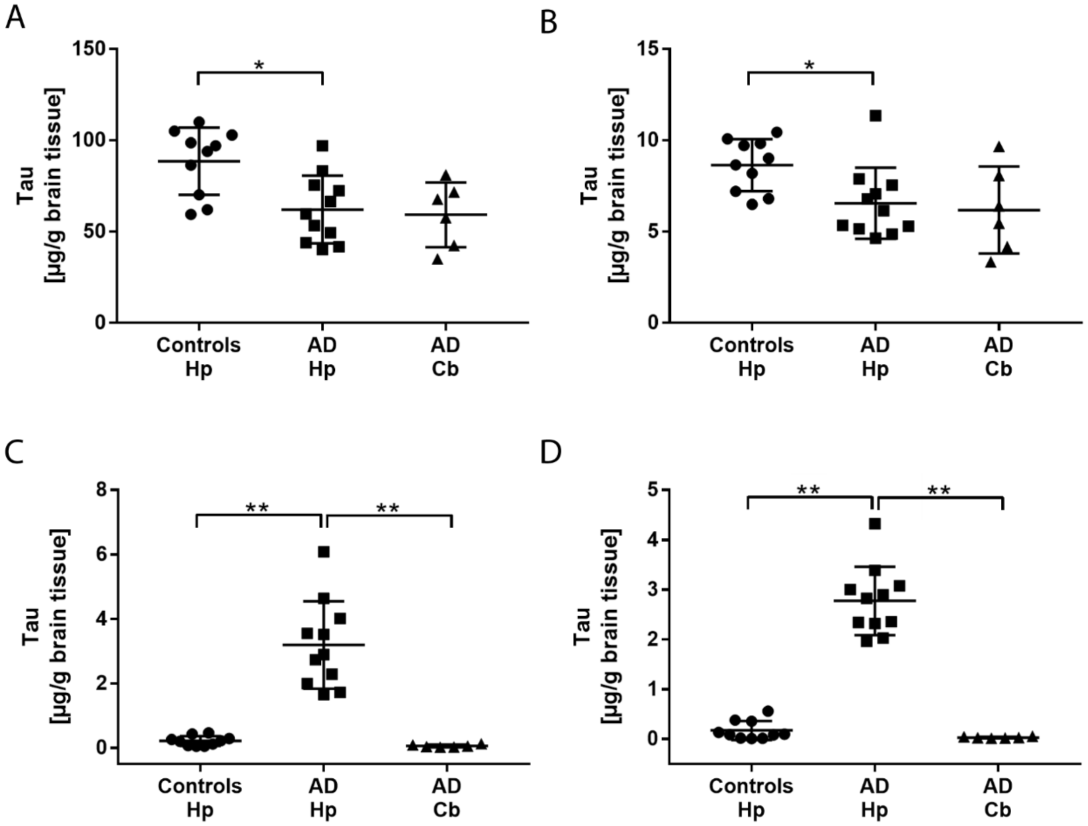

Figure S4 – Total Tau levels are increased in the insoluble fraction of AD hippocampus. Total Tau levels were measured by Tau12/HT7 ELISA in the sarkosyl-soluble SS1 (**A**) and S3 (**B**) fractions and in the sarkosyl-insoluble PS1 (**C**) and P3 (**D**) extracts of human brain samples. Levels of Tau are lower in the soluble SS1 and S3 extracts of human AD than control hippocampus (p=0.006 for SS1 and p=0.036 for S3). Tau is similar in AD hippocampus and cerebellum (p=0.937 for SS1 and p=0.758 for S3). In the insoluble PS1 and P3 extracts, Tau levels are significantly higher in AD hippocampus than control hippocampus (p<0.0001 for PS1 and p=0.002 for P3) and AD cerebellum (p<0.0001 for PS1 and p=0.002 for P3). In all graphs, data points, mean and standard deviation are shown. AD hippocampus, n=11; AD cerebellum, n=6; Control hippocampus, n=10. Hp, hippocampus; Cb, cerebellum. *p<0.01; **p<0.01

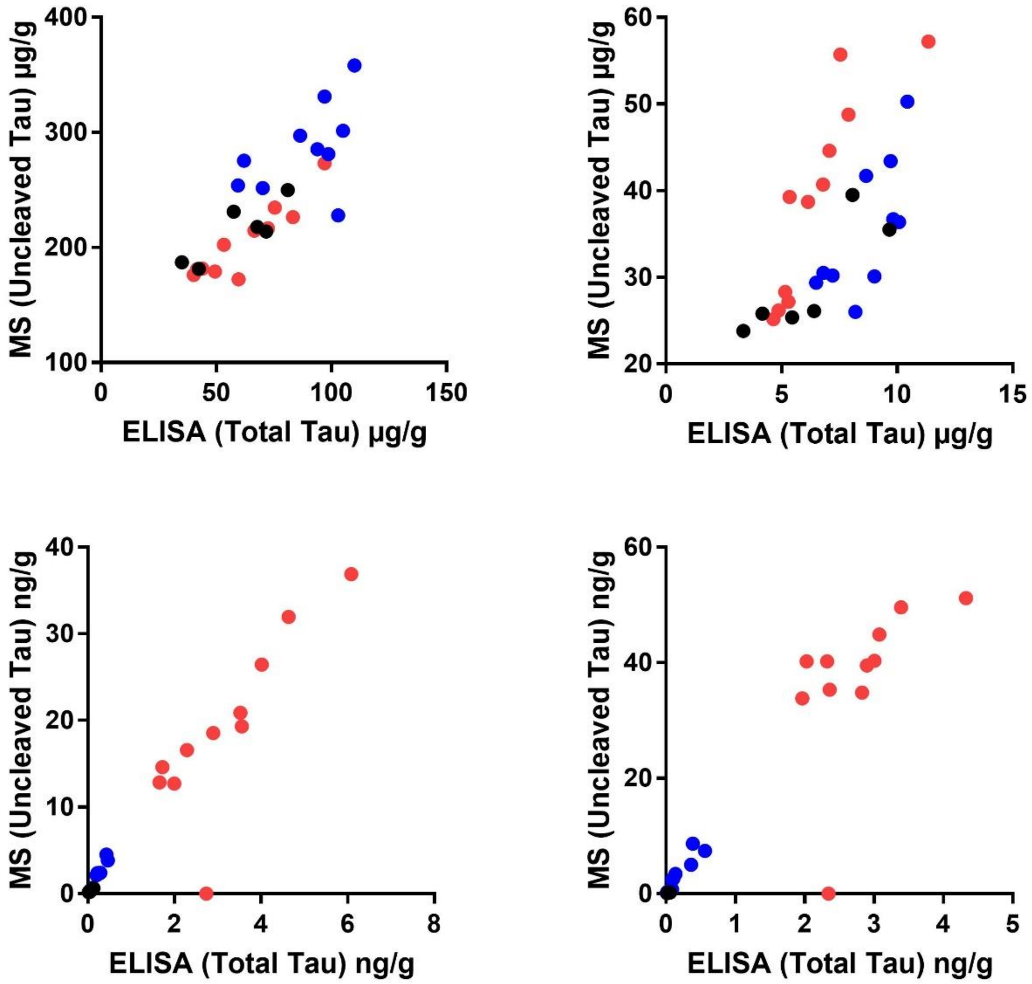

Figure S5 – Total Tau levels measured by ELISA correlates with levels of uncleaved Tau determined by LC-MS/MS. Correlations are shown for measurements in the sarkosyl-soluble SS1 (A) and S3 (B) fractions and in the sarkosyl-insoluble PS1 (C) and P3 (D) extracts of human brain samples. Symbol colors represent AD hippocampus, red; Control hippocampus, blue; AD cerebellum, black.

Figure S6: Fragment spectra of the experiment shown in Figure 1

Band B1* - N368/K369

Band 2 - N255/V256

Band 2 - N255/V256, Oxidized Methionine

Band B3 - N167/A168 Deaminated Asparagine

Band B3 - N167/A168

Band B4 - N368/K369

Band B5 – N167/1A68

Band B5 - N255/V256, Oxidized Methionine

Band B6 – N296/I297

Band B1*

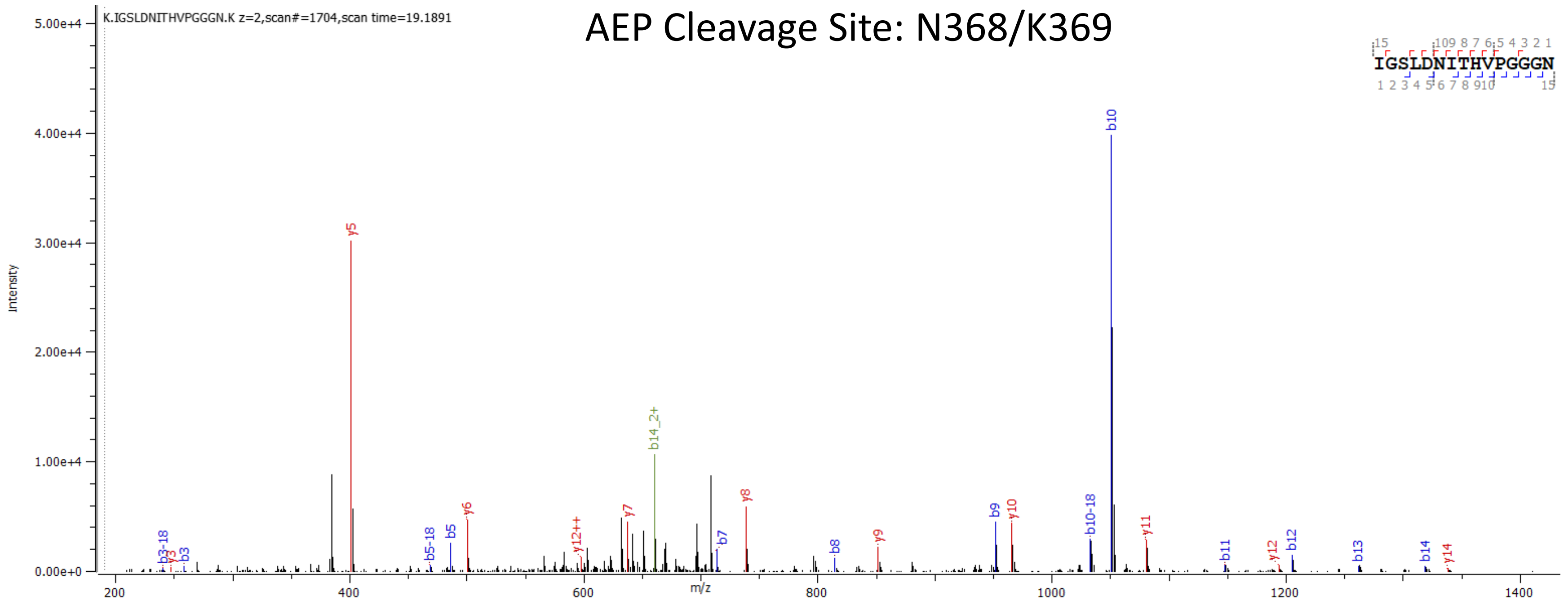

K.IGSLDNITHVPGGGN.K z=2,scan#=1704,scan time=19.1891
AEP Cleavage Site: N368/K369
IGSLDNITHVPGGGN
Intensity
m/z
5.00e+4
4.00e+4
3.00e+4
2.00e+4
1.00e+4
0.00e+0
200
400
600
800
1000
1200
1400
b10
y5
b14_2+
y8
y10
b9
b10-18
y11
y7
y6
b5
b5-18
y12++
b7
y9
b8
b3-18
y3
b3
b11
y12
b12
b13
b14
y14

Band B2

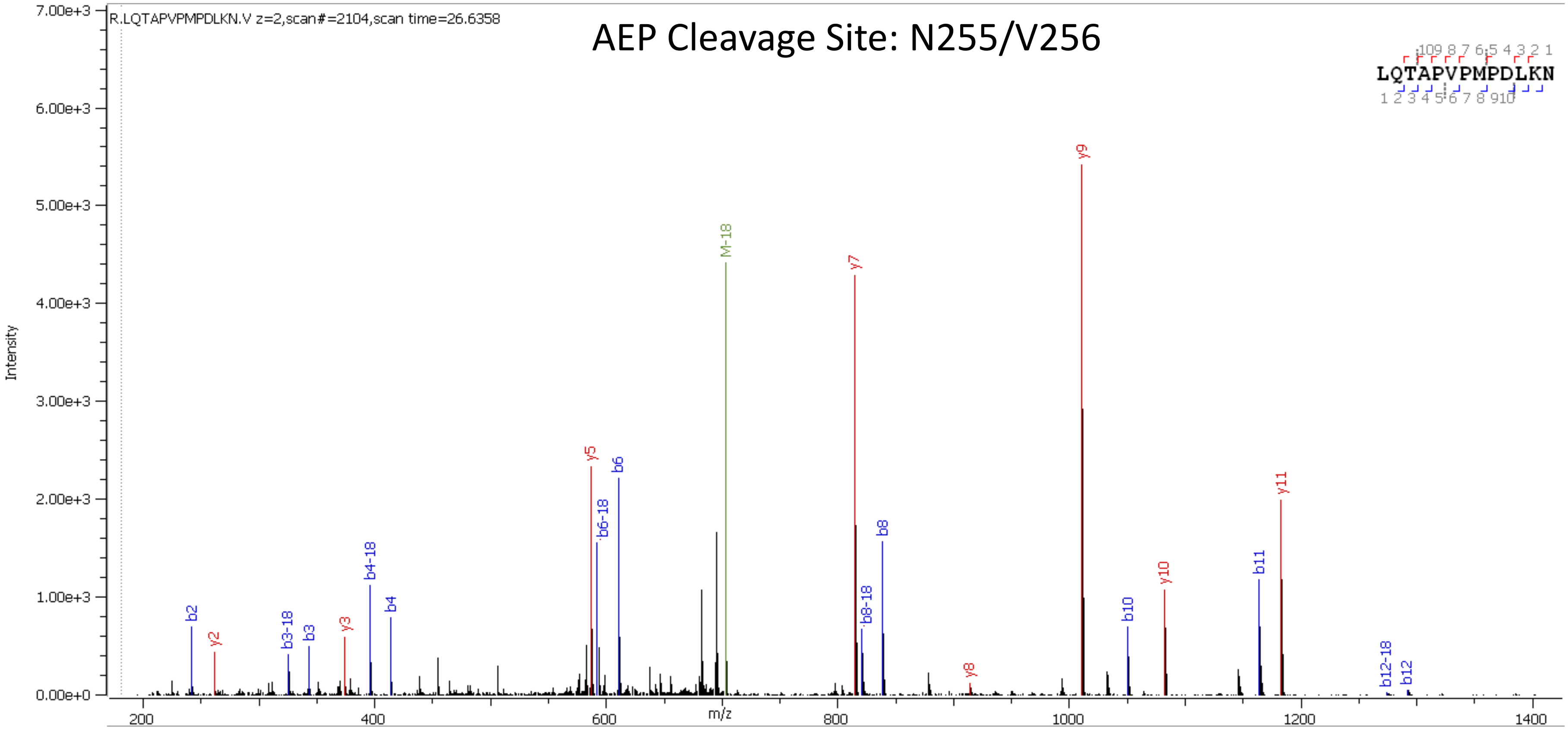

R.LQTAPVPMPDLKN.V z=2,scan#=2104,scan time=26.6358
AEP Cleavage Site: N255/V256
LQTAPVPMPDLKN
Intensity
m/z

Band B2

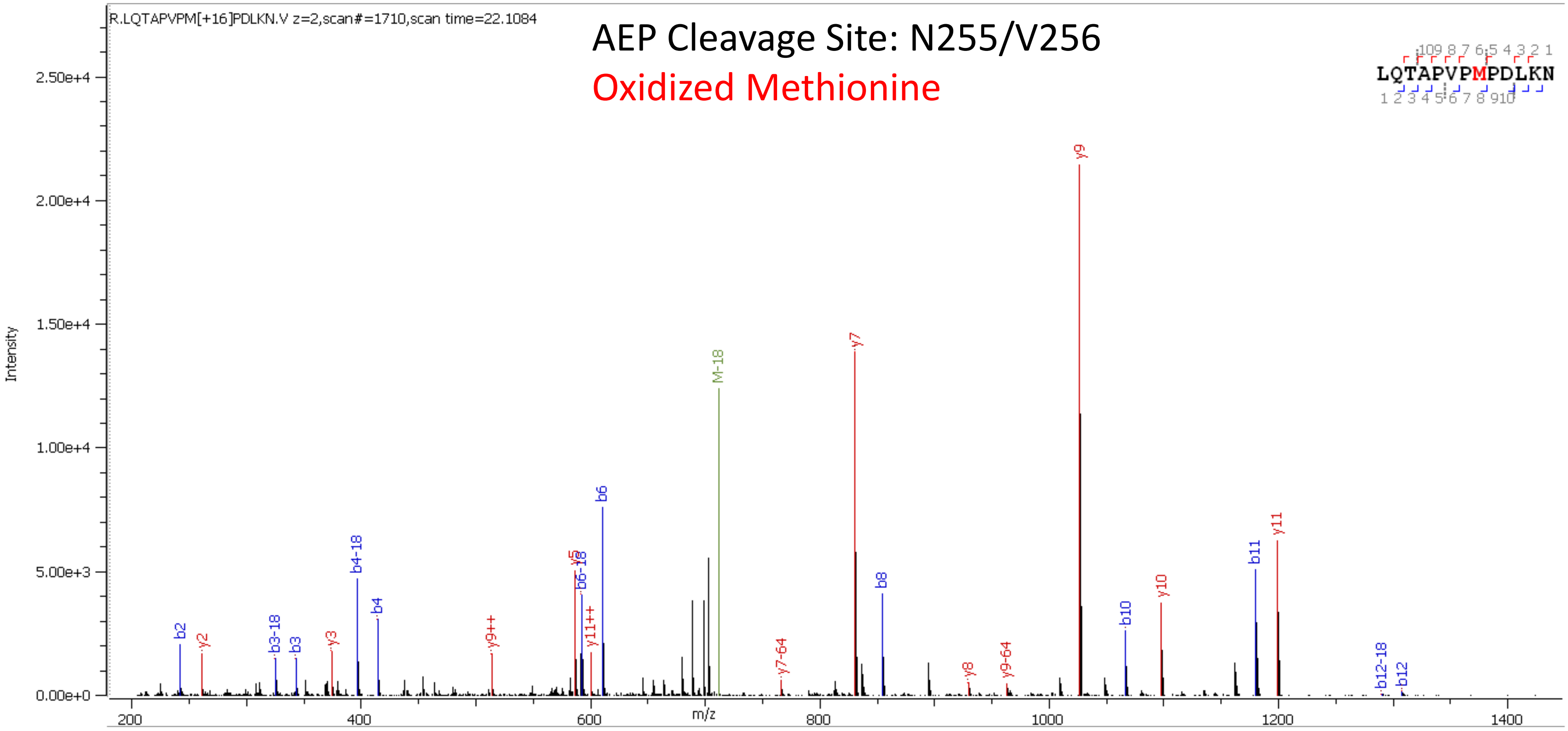
R.LQTAPVPM[+16]PDLKN.V z=2,scan#=1710,scan time=22.1084
AEP Cleavage Site: N255/V256
Oxidized Methionine
LQTAPVPMPDLKN
Intensity
m/z
2.50e+4
2.00e+4
1.50e+4
1.00e+4
5.00e+3
0.00e+0
200
400
600
800
1000
1200
1400
b2
y2
b3-18
b3
y3
b4-18
b4
y9++
y5
b6-18
y11++
b6
M-18
y7-64
y7
b8
y8
y9-64
y9
b10
y10
b11
y11
b12-18
b12

Band B3

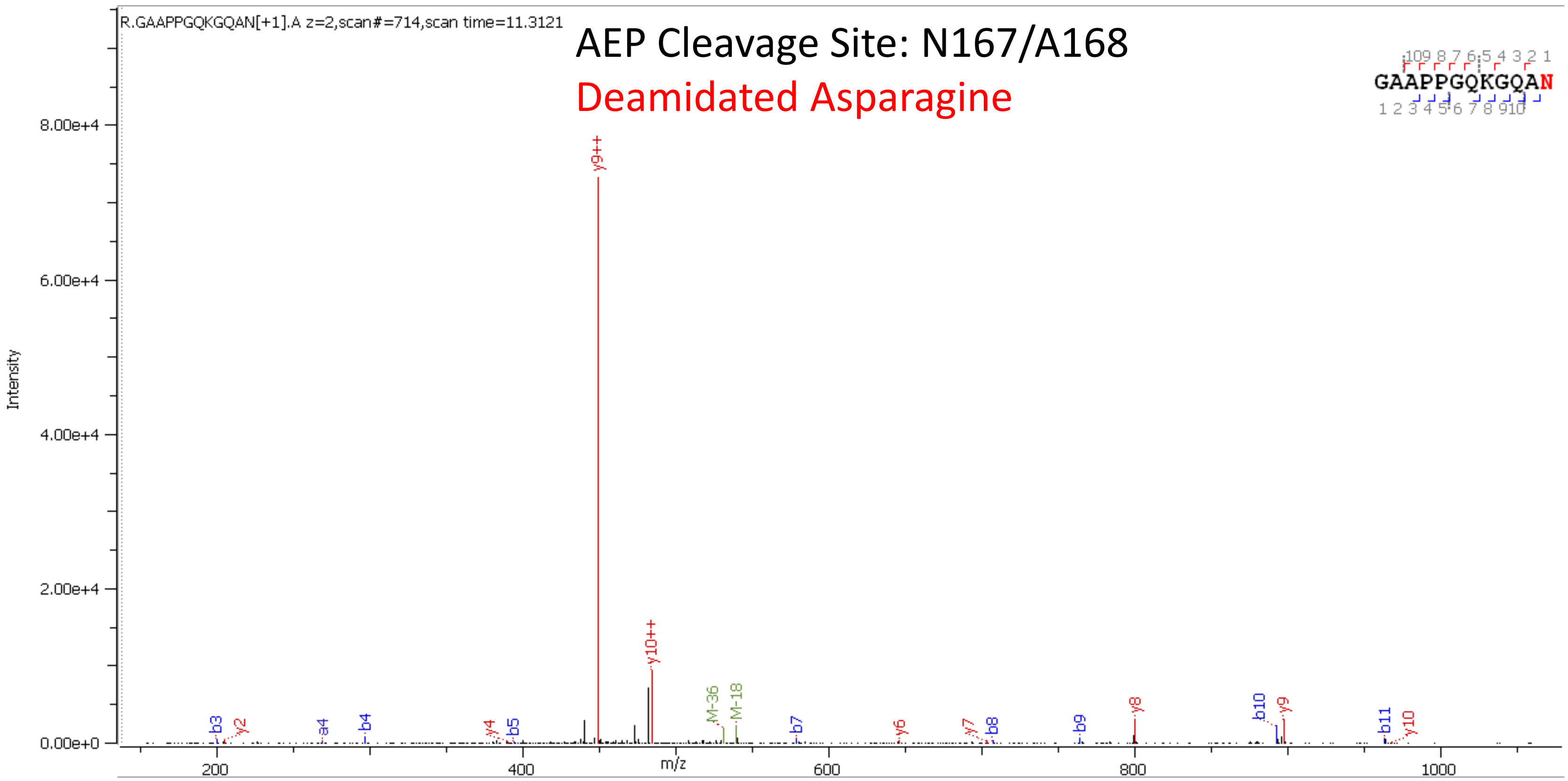
R.GAAPPGQKGQAN[+1].A z=2,scan#=714,scan time=11.3121
AEP Cleavage Site: N167/A168
Deamidated Asparagine
GAAPPGQKGQAN
Intensity
m/z
y9++
y10++
M-36
M-18
b3
y2
a4
b4
y4
b5
b7
y6
y7
b8
b9
y8
b10
y9
b11
y10
0.00e+0
2.00e+4
4.00e+4
6.00e+4
8.00e+4
200
400
600
800
1000

Band B3

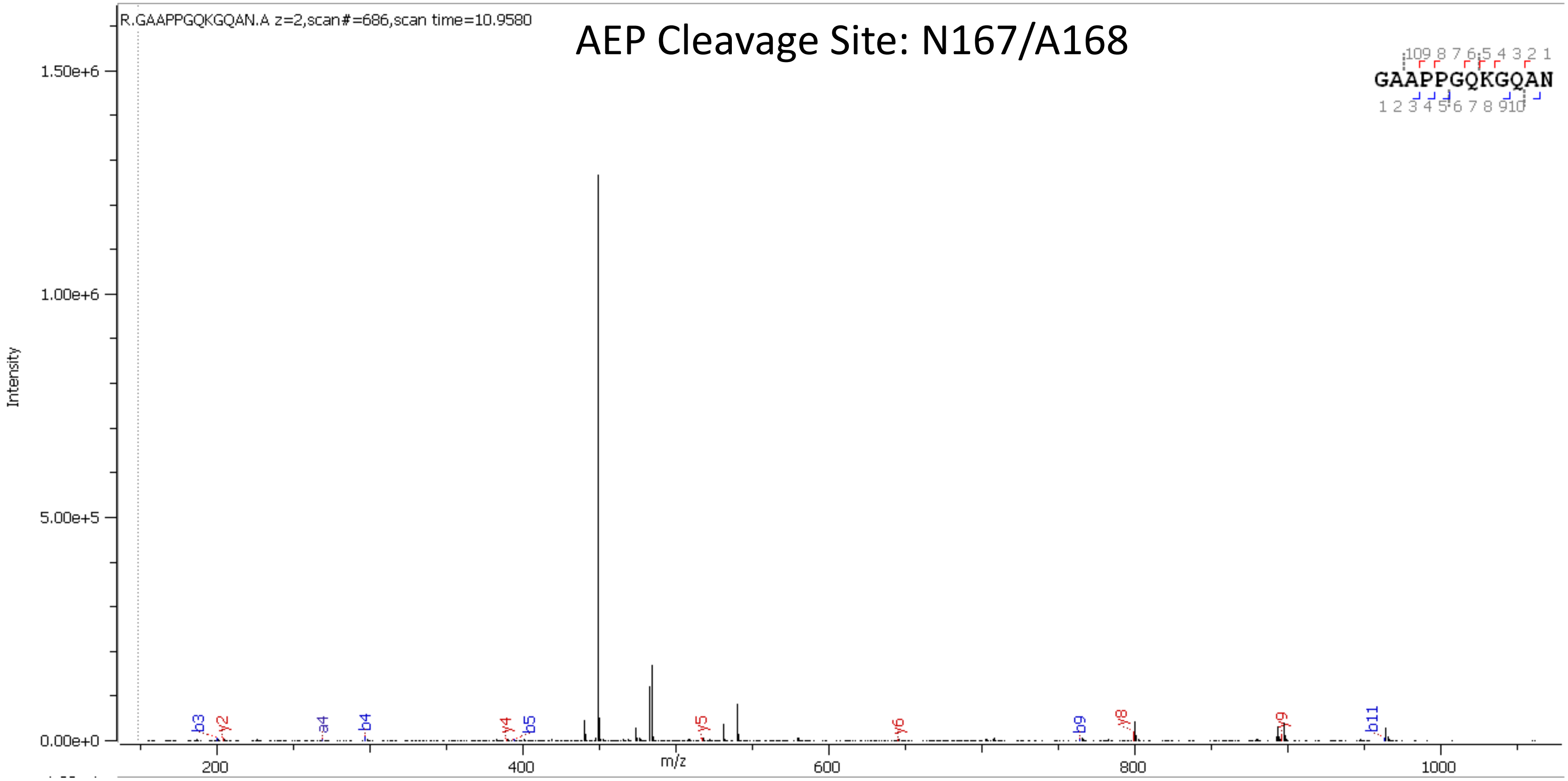
R.GAAPPGQKGQAN.A z=2,scan#=686,scan time=10.9580
AEP Cleavage Site: N167/A168
GAAPPGQKGQAN
Intensity
m/z
1.50e+6
1.00e+6
5.00e+5
0.00e+0
200
400
600
800
1000
b3
y2
a4
b4
y4
b5
y5
y6
b9
y8
y9
b11

Band B4

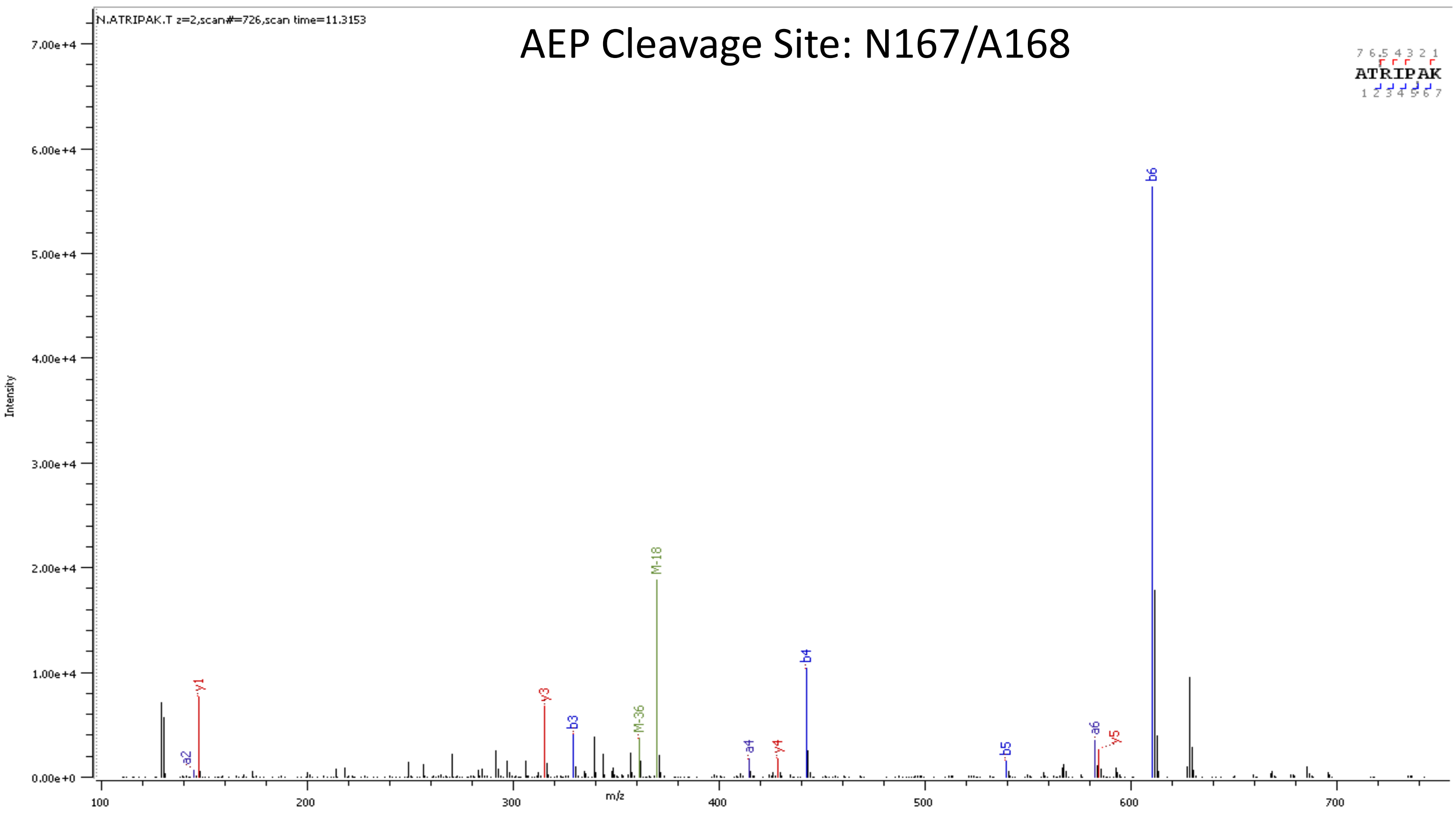

N.ATRIPAK.T z=2,scan#=726,scan time=11.3153
AEP Cleavage Site: N167/A168
ATRIPAK
Intensity
m/z
b6
M-18
b4
y1
y3
b3
M-36
a2
a4
y4
b5
a6
y5

Band B4

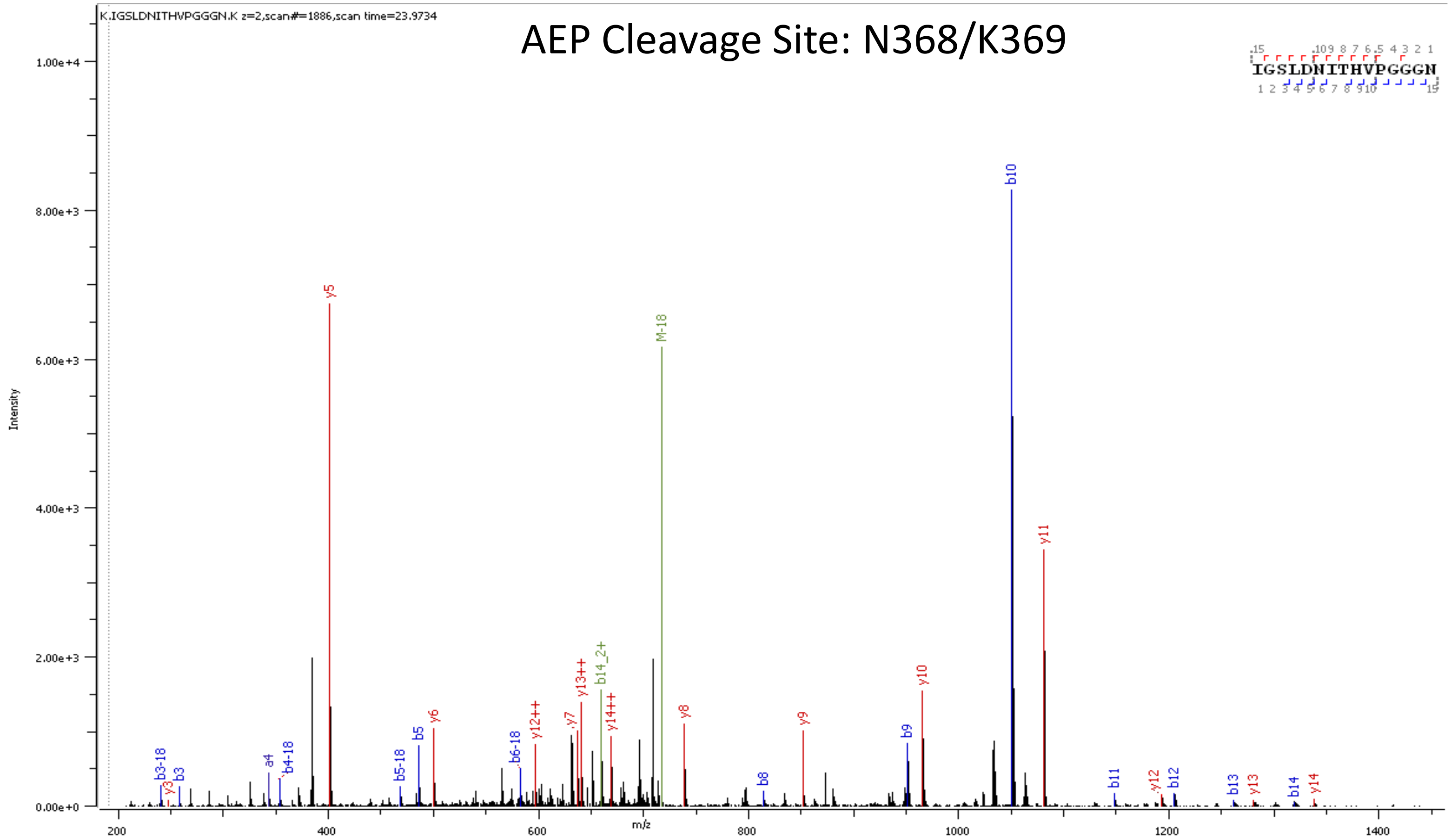
K.IGSLDNITHVPGGGN.K z=2,scan#=1886,scan time=23.9734
AEP Cleavage Site: N368/K369
IGSLDNITHVPGGGN
Intensity
m/z
b10
y5
M-18
y11
b14_2+
y13++
y10
y14++
y12++
y6
y8
y9
b9
y7
b5
b6-18
b5-18
b4-18
a4
b3-18
b3
y3
b8
b11
y12
b12
b13
y13
b14
y14

Band B5

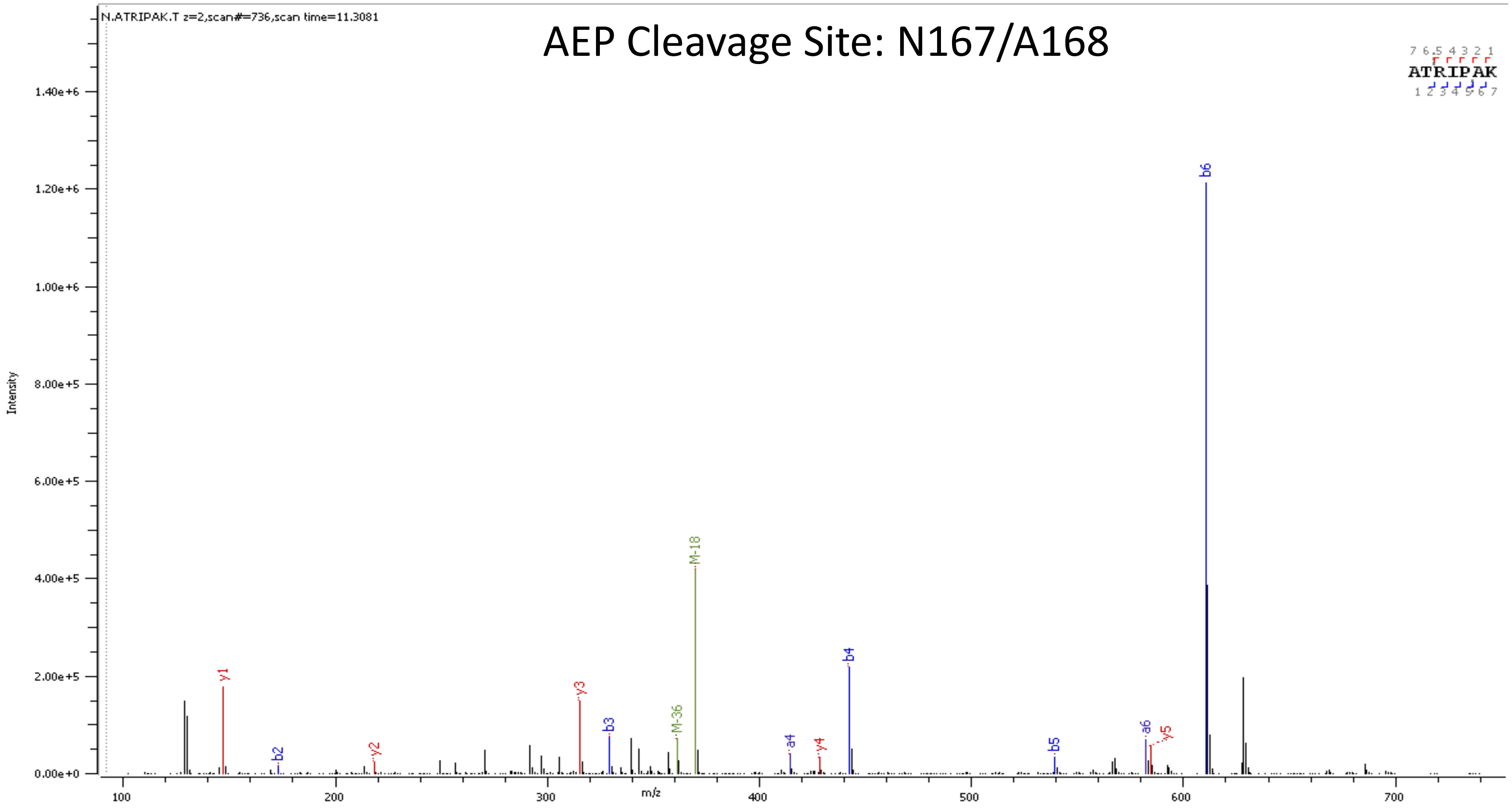
N.ATRIPAK.T z=2,scan#=736,scan time=11.3081
AEP Cleavage Site: N167/A168
7 6 5 4 3 2 1
ATRIPAK
1 2 3 4 5 6 7
b6
M-18
b4
y1
y3
b3
M-36
a4
y4
b5
a6
y5
b2
y2
Intensity
m/z
1.40e+6
1.20e+6
1.00e+6
8.00e+5
6.00e+5
4.00e+5
2.00e+5
0.00e+0
100
200
300
400
500
600
700

Band B5

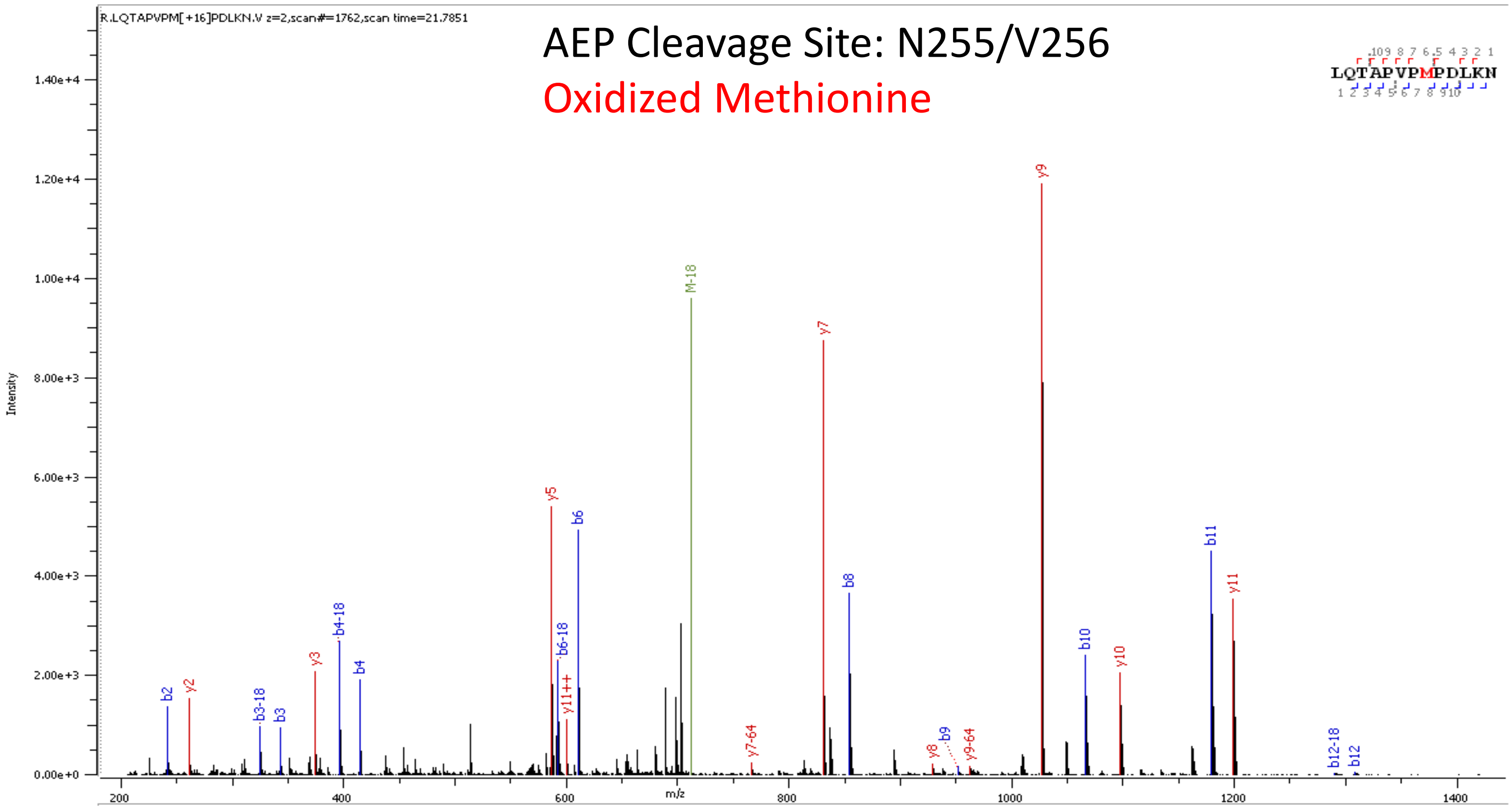
R.LQTAPVPM[+16]PDLKN.V z=2,scan#=1762,scan time=21.7851
AEP Cleavage Site: N255/V256
Oxidized Methionine
LQTAPVPMPDLKN
Intensity
m/z
1.40e+4
1.20e+4
1.00e+4
8.00e+3
6.00e+3
4.00e+3
2.00e+3
0.00e+0
200
400
600
800
1000
1200
1400
b2
y2
b3-18
b3
y3
b4-18
b4
y5
b6-18
y11++
b6
M-18
y7-64
y7
b8
y8
b9
y9-64
y9
b10
y10
b11
y11
b12-18
b12

Band B6

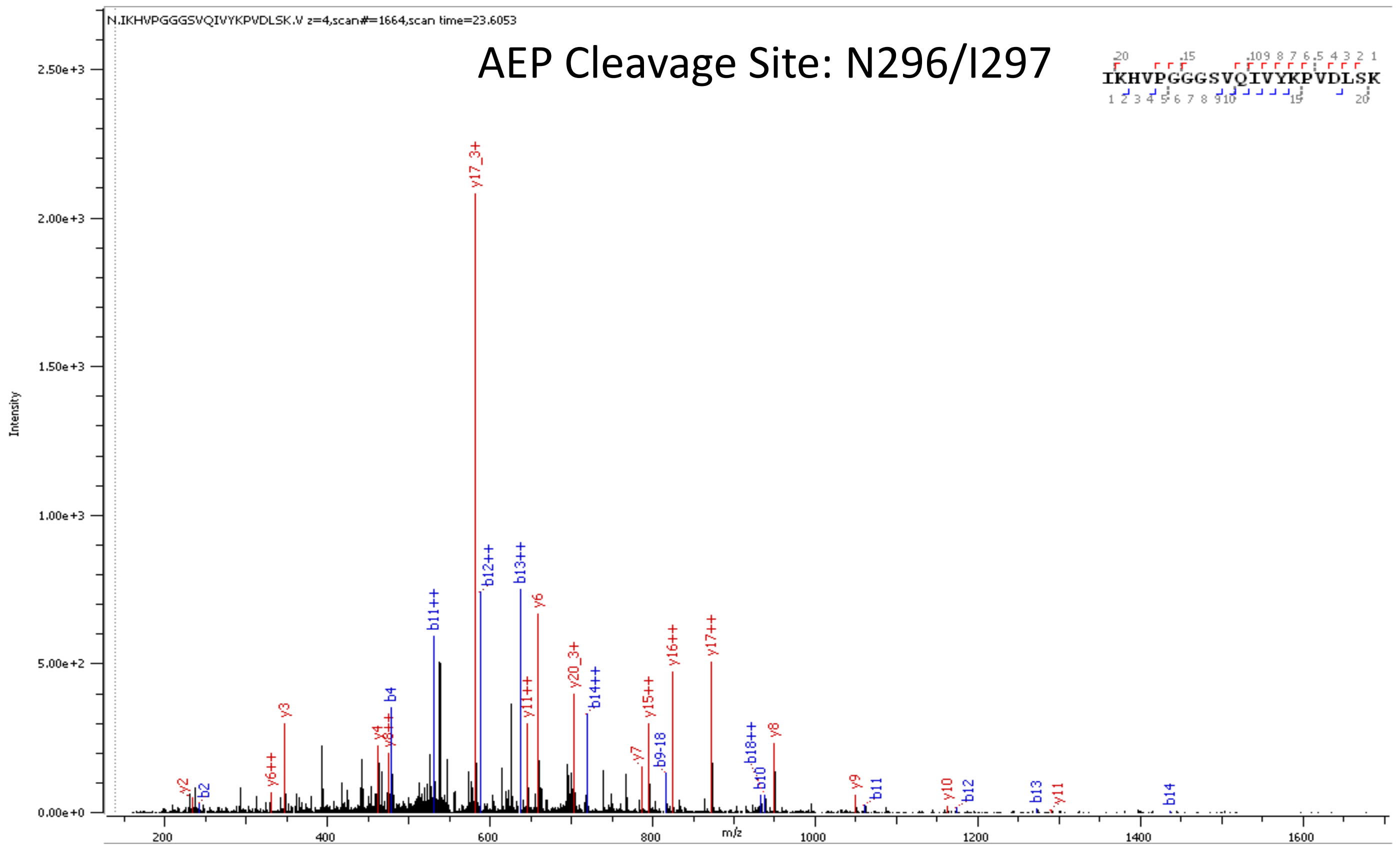

N.IKHVPGGGSVQIVYKPVDLSK.V z=4,scan#=1664,scan time=23.6053
AEP Cleavage Site: N296/I297
IKHVPGGGSVQIVYKPVDLSK
Intensity
m/z
